# Monitoring membrane viscosity in differentiating stem cells using BODIPY-based molecular rotors and FLIM

**DOI:** 10.1038/s41598-020-70972-5

**Published:** 2020-08-20

**Authors:** Alena S. Kashirina, Ismael López-Duarte, Markéta Kubánková, Alexander A. Gulin, Varvara V. Dudenkova, Svetlana A. Rodimova, Hayk G. Torgomyan, Elena V. Zagaynova, Aleksandra V. Meleshina, Marina K. Kuimova

**Affiliations:** 1 Privolzhsky Research Medical University, 10/1 Minin and Pozharsky Sq., Nizhny Novgorod, Russian Federation 603950; 2 Department of Chemistry, Imperial College London, Molecular Sciences Research Hub, White City Campus, London, W12 0BZ UK; 3 N.N. Semenov Federal Research Center for Chemical Physics, Russian Academy of Sciences (FRCCP RAS), Kosygin st. 4, Moscow, Russian Federation 119991; 4 Department of Chemistry, Lomonosov Moscow State University, Leninskiye Gory 1-3, Moscow, Russian Federation 119991; 5 Lobachevsky State University of Nizhny Novgorod, 23 Gagarin Avenue, Novgorod, Nizhny Novgorod, Russian Federation 603950

**Keywords:** Stem cells, Mesenchymal stem cells, Cellular imaging

## Abstract

Membrane fluidity plays an important role in many cell functions such as cell adhesion, and migration. In stem cell lines membrane fluidity may play a role in differentiation. Here we report the use of viscosity-sensitive fluorophores based on a BODIPY core, termed “molecular rotors”, in combination with Fluorescence Lifetime Imaging Microscopy, for monitoring of plasma membrane viscosity changes in mesenchymal stem cells (MSCs) during osteogenic and chondrogenic differentiation. In order to correlate the viscosity values with membrane lipid composition, the detailed analysis of the corresponding membrane lipid composition of differentiated cells was performed by time-of-flight secondary ion mass spectrometry. Our results directly demonstrate for the first time that differentiation of MSCs results in distinct membrane viscosities, that reflect the change in lipidome of the cells following differentiation.

## Introduction

Membrane fluidity is considered a key parameter influencing biological function of cells, such as cell adhesion, migration and differentiation^1^. These properties are of particular importance in stem cell lines, where small modifications in membrane parameters have the potential to either promote a lineage commitment or a self-renewal^2^. The plasma membrane is the interface between a cell and its environment, it directly interacts with the matrix outside the cell and is responsible for many important tasks such as signaling and mass transfer. In stem cells, its composition and properties are likely to reflect their differentiation status.

However, little is known on how the viscosity parameters of different stem cell lineages can change depending on the direction of differentiation. There is evidence that the membrane fluidity substantially changes during induced pluripotent stem cells (iPS) differentiation. Generalized polarization monitoring was previously used to detect the rise of membrane rigidity during iPSC differentiation^1^. Furthermore, the results in^1^ potentially suggested that membrane rigidification could be transmitted to neighboring cells, resulting in the acceleration of a cells differentiation, in a wave-line fashion. It was also reported that the viscoelastic properties can predict which subpopulations of undifferentiated mesenchymal stem cells (MSCs) differentiate into osteocytes, and which would turn into adipocytes or chondroblasts. The stiffest cell populations produced more bone cells; the softest cells predominantly produced fat cells; the cells with the highest viscosity became cartilage cells^3^. While cell stiffness measured in^3^ is a distinctly different property to the cell membrane viscosity, both depend on membrane lipid compositions. There is evidence that differentiation of human mesenchymal stem cells (MSCs) into osteoblasts, chondrocytes or adipocytes produces specific membrane compositions and biophysical properties, based on mass spectrometry data^2,4^. However, to the best of our knowledge, there is no direct measurements of viscosity in the plasma membrane of differentiated MSCs.

Such measurements are not trivial, since traditional mechanical methods for probing viscosity are not suitable for living cells due to their invasiveness and necessity of large volumes of sample for analysis. However, several spectroscopic and microscopic approaches to monitor viscosity through diffusion rates have already been developed. These include fluorescence recovery after photobleaching (FRAP)^5^, fluorescence correlation spectroscopy (FCS)^6–8^ or single particle tracking^9^. Here we report the use of fluorescence lifetime imaging microscopy (FLIM) and viscosity-sensitive fluorophores termed “molecular rotors” as an attractive approach that allows the direct measurement of the plasma membrane viscosity in cell culture in a non-destructive manner, while analyzing a large number of cells at the same time^10–14^.

Fluorescent molecular rotors are small synthetic fluorophores, in which excited state deactivation is strongly depends on the viscosity of the immediate environment of the rotor. In order to obtain quantitative viscosity measurements with such rotors it is necessary to first correlate the rotor’s fluorescence lifetime to viscosity independently, in well-defined calibration systems of known viscosity. Following calibration, the rotor can be incorporated in an environment of unknown viscosity and its lifetime measured using FLIM. Large number of cells can be analyzed at the same time (in a field of view of a confocal or multiphoton FLIM), overcoming the limitation of most spectroscopic methods that can determine the mobility only in single points at any one time (e.g. FCS, FRAP). We have previously successfully applied FLIM with molecular rotors to measure the plasma membrane viscosity, using the rotors that can be targeted to the plasma membrane^10–12^.

4,4-Difluoro-4-bora-3a,4a-diaza-s-indacenes (BODIPYs) are a popular class of fluorescent probes that is characterized by high extinction coefficients, quantum yields and photostability^15^. BODIPY-based molecular rotors have been successfully used in the past for quantitative viscosity imaging in 2D and 3D cell culture^16^ and in vivo^17^ and can be targeted to the plasma membranes^10–12^. Interestingly, there is a large variation in plasma membrane viscosity determined from different mammalian cell types^10,18^, from 100 to 1,000 cP at 37 °C. This data lends grounds for our hypothesis that MSCs cell differentiation can lead to large variations in plasma membrane viscosity.

The current work was aimed at monitoring the membrane viscosity changes in MSCs during differentiation to osteogenic and chondrogenic lineages. The membrane viscosity was determined from viscosity-dependent fluorescence lifetimes of a charged membrane-targeting molecular rotor based on BODIPY structure, using FLIM. Additionally, to correlate the observed viscosity values with membrane lipid composition, the detailed analysis of the corresponding membrane lipid composition of differentiated cells was performed by time-of-flight secondary ion mass spectrometry (ToF–SIMS).

## Results

### Osteogenic and chondrogenic differentiation of MSCs

As shown previously with the use of flow cytometry, the MSCs had the phenotype of normal mesenchymal cells and expressed the typical markers: more than 77.5% of cells expressed CD105, CD90 and CD73, while less than 22% expressed the negative surface markers CD34, CD45, HLA-DR, CD11b or CD19^21^.

**Figure 1:**
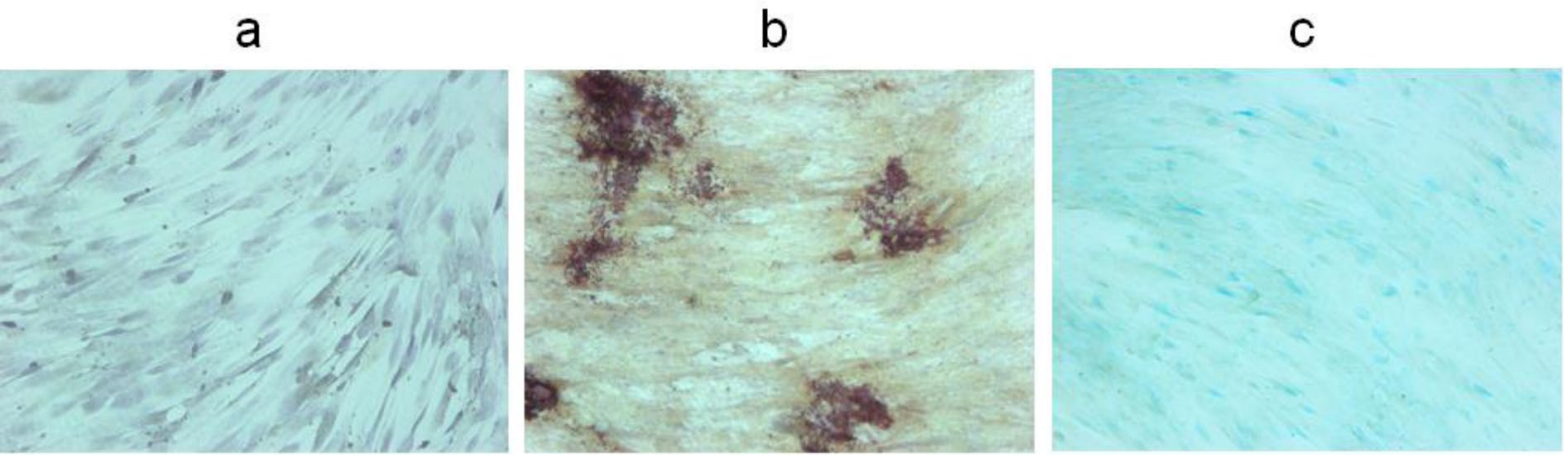
Microscopic images of MSCs in transmitted light. Undifferentiated cells (**a**), incubated in standard growth medium on day 0 of culturing (*Hematoxylin* and *Eosin* staining); on day 21 of osteogenic differentiation (Alizarin Red S staining) (**b**); on day 21 of chondrogenic differentiation (Alcian blue staining) (**c**). The image size is 1,289 × 964 μm.

MSC differentiation was identified by cellular morphology and specific staining. Before differentiation the MSCs population was homogeneous and the cells had a fusiform morphology, which was confirmed by Hematoxylin and Eosin staining (Fig. 1a). By the 21st day of differentiation, the cells acquired a polygonal shape. Confirmation of the differentiation of MSCs was carried out by staining with Alizarin Red S, which showed the calcification of the extracellular matrix in the case of osteogenic differentiation and Alcian blue for the presence of glycosaminoglycans in the case of chondrogenic differentiation (Fig. 1b,c). The cells which were not incubated in differentiating media retained their fusiform morphology through to day 21 and did not show any specific staining with the corresponding dyes (SI, Fig. S6). However, it should be noted that undifferentiated MSCs on day 21 of culturing were growing in a significantly more dense monolayer, as compared to 0 days.

### The choice of molecular rotor and viscosity analysis of the plasma membrane of MSCs during differentiation by FLIM

**Scheme 1:**
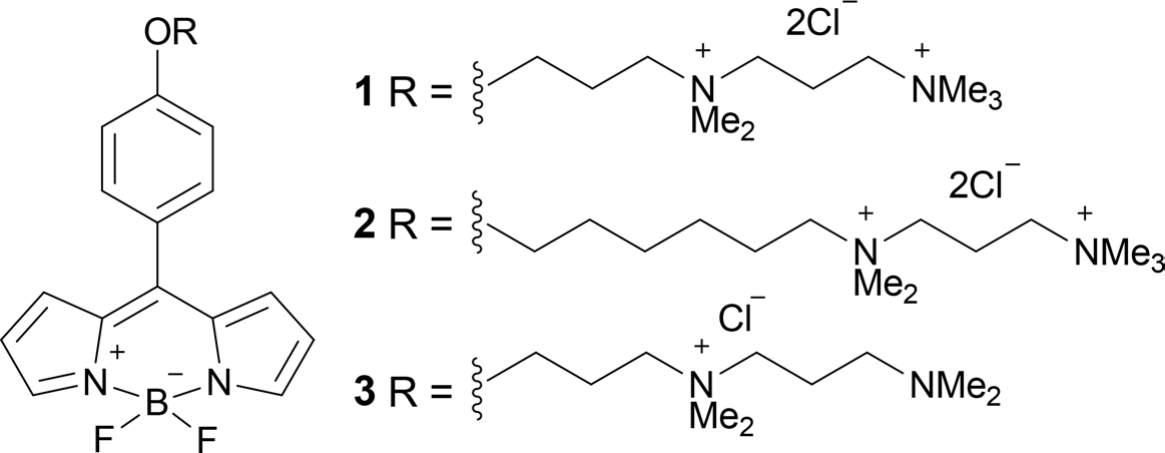
Chemical structure of BODIPYs.

We initially attempted to stain undifferentiated and differentiated MSCs with the previously reported molecular rotor BODIPY 2, that was successfully used in 2D and 3D cell culture and in vivo^16,17^. However, poor cell staining was observed for this rotor (SI, Fig. S7). Higher concentrations of BODIPY 2 in the staining medium resulted in brighter images, however, it was clear from the decay analysis that the lifetimes obtained from cellular membranes change significantly depending on the incubation concentration, which is a signature of the dye aggregation (SI, Fig. S7, 8)^22^. Poor staining of membranes might be due to an insufficient solubility of BODIPY 2, which might affect its availability to stain the densely packed cell monolayer at later stages of differentiation. Therefore, to further enhance aqueous solubility of the rotor, we have synthesized a new derivative, BODIPY 1 (see Scheme 1 for the structures).

Compared to the rotor used in a recent study by Sherin et al., BODIPY 3, the new rotor reported here has two positive charges on its short C_3_ chain, which should further enhance its aqueous solubility.

The calibration of the new BODIPY 1 rotor was performed in glycerol at a range of temperatures, 1.2–100 °C (SI, Fig. S4). A large dynamic range of fluorescence lifetimes between 0.1 and 5.57 ns was observed in response to the viscosity change between 1 and 12,000 cP. The data can be fitted by the following equation (SI, Fig. S5):

1 $${\text{y}} = {\text{Vmax}}*{\text{x}}^{{\text{n}}} {/}({\text{k}}^{{\text{n}}} + {\text{x}}^{{\text{n}}} )$$

where x is viscosity in cP and y is the fluorescence lifetime in ps. The parameters of the fit (Vmax, k, n) are given in Fig. S5, ESI. From our previous data for a large family of BODIPY rotors we know that these dyes successfully incorporate in the tail region of lipid bilayers, both in model lipid bilayers of various composition^22^ and in live cell plasma membranes of bacterial and mammalian cells^10,13,14,17^. We also previously verified that their calibration curves are polarity and temperature-independent^10,11^, making the current glycerol-derived calibration curve suitable for the plasma membrane viscosity measurements.

**Figure 2:**
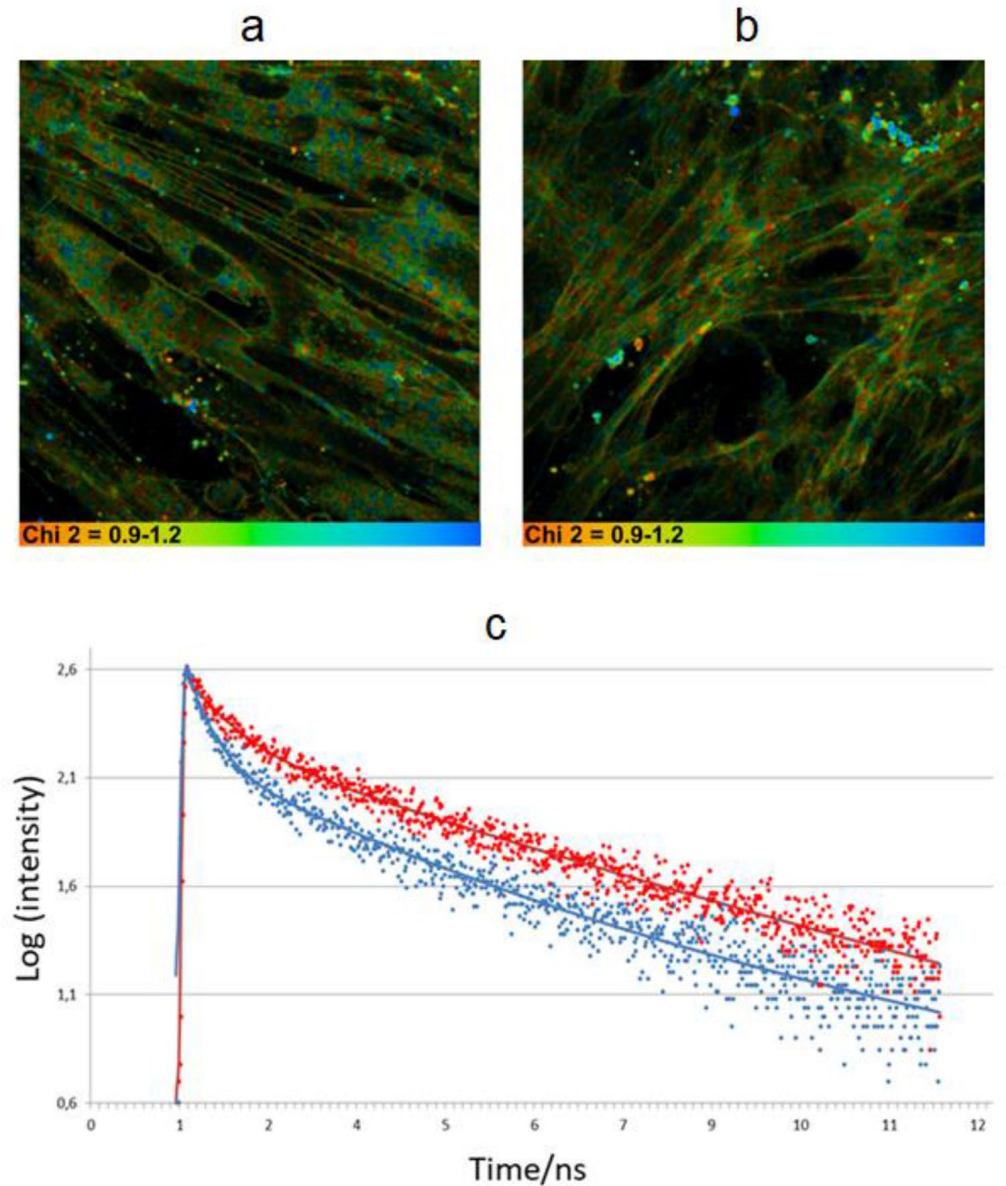
FLIM of undifferentiated (**a**) and chondrogenically differentiated MSCs (**b**) on day 21 of cell culture; the distribution of χ^2^ for the bi-exponential fitting model is shown. (**c**) Typical point decays together with bi-exponential fits for undifferentiated cells (blue, fitted parameters: τ1 = 514.45 ps, τ2 = 4182.2 ps, α1 = 66.55%) and differentiated cells (red: τ1 = 594.14 ps, τ2 = 4187.3 ps, α1 = 63.57%); χ^2^ was 1.2 for both traces. (SPCImage 5.7, https://www.becker-hickl.com).

Undifferentiated and differentiated MSC cells were successfully stained with BODIPY 1. Both the plasma membrane staining and the dye internalization was detected in all cells studied. It was impossible to automatically separate the cell cytoplasm and the plasma membrane of each cell for the data analysis, and, therefore, the decays in the region corresponding to the plasma membrane were selected and analyzed by hand (ca. 30 locations for each image, to accumulate good statistics). The lifetimes of internalized BODIPY 1 were not analyzed, due to a high likelihood of aggregation in these locations (indicated by high χ^2^ values). FLIM analysis in the membrane locations showed biexponential decay kinetics with χ^2^ mainly in the range of 0.9–1.2 both in control and differentiated MSCs (Fig. 2).

It is well known that for BODIPY-based dyes the presence of bi-exponential decay can be indicative of dye aggregation, with aggregated species characterized by a weak emission band centered at 650–700 nm^17^. Aggregate formation results in the quenching of the main emission band centered at 515 nm, which renders the lifetime-viscosity calibration curve unusable. However, occasionally, the biexponential decay of BODIPY rotors in lipid bilayers can also indicate the presence of extremely viscous lipid phases in the plasma membrane, such as liquid ordered phases^11^. This behavior was observed in the liquid ordered (L_o_) phases in phase-separated vesicles^11^, in plasma membrane of live *E. coli* cells^13^ and in the eye lens cell membranes^10^.

To verify whether the biexponential decays observed in the present case of MSC cells are due to aggregation or the presence of ordered lipid phases, several control experiments were performed. Firstly, it is known that due to the high metabolic activity and high levels of NAD(P)H present, MSCs are characterized by a significant autofluorescent signal, which can contribute to the biexponential decay kinetics observed. Considering this, we tested the presence of autofluorescence in MSCs membranes and verified that at our experimental conditions (i.e. dye loading and incubation times), autofluorescence does not contribute significantly (SI, Fig. S9) in the plasma membrane region of cells, which is of interest for this study. Secondly, we recorded FLIM for BODIPY 1 in undifferentiated and differentiated MSCs in the two spectral windows, for monomers and aggregates (500–550 nm and 598–660 nm, respectively). Analysis showed that the decays were identical (SI, Fig. S10), thus confirming that the aggregation of BODIPY 1 was not contributing to the observed decays.

**Figure 3:**
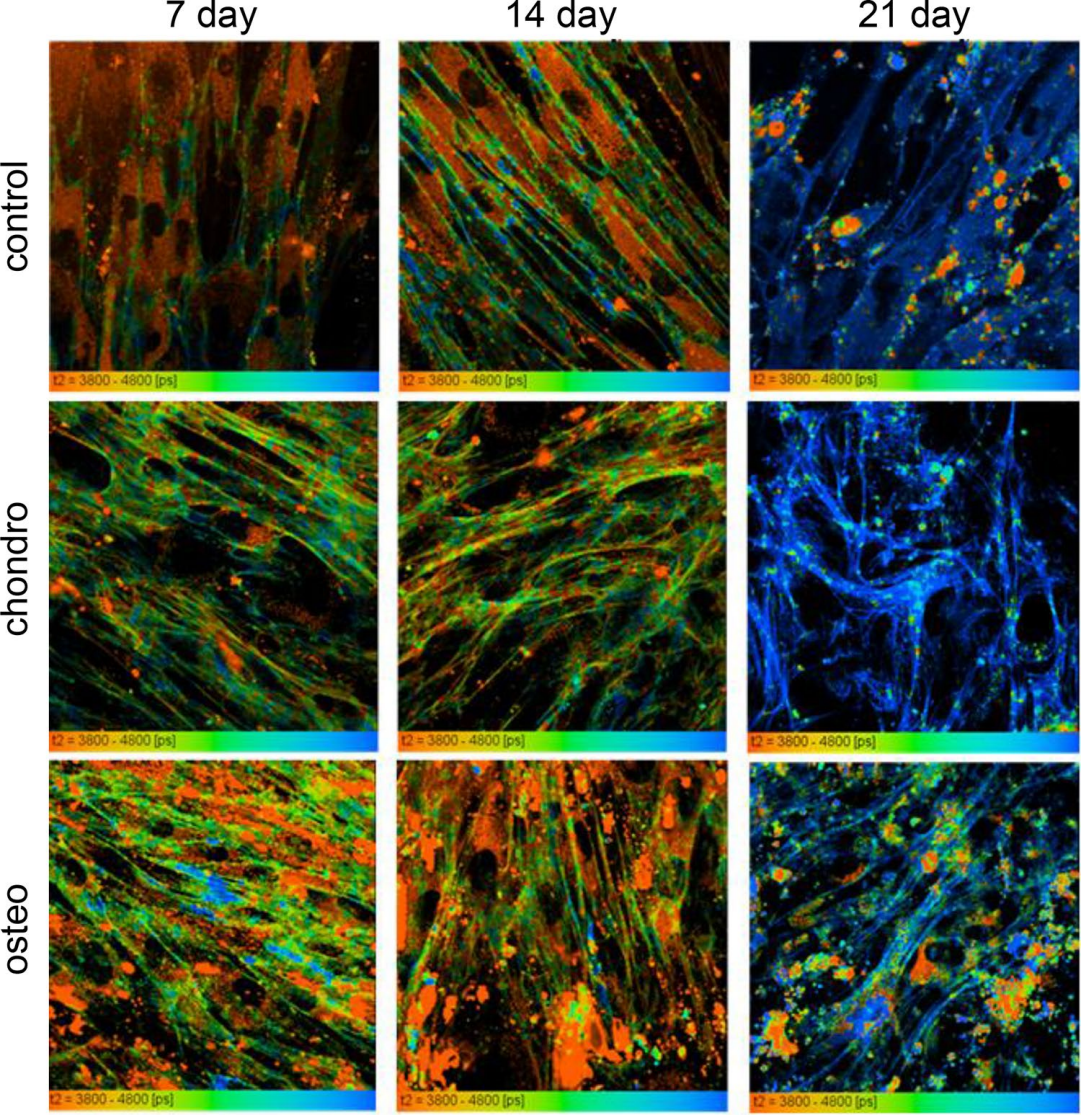
FLIM of MSCs, stained with BODIPY 1 during osteogenic (bottom) and chondrogenic (middle) differentiations compared to undifferentiated control (top row), on days 7, 14 and 21; pseudocolor-coded images of the long (τ2) component are shown between 3.8 and 4.8 ns, which correspond to the viscosity of the lipid tail region of the bilayer. Field of view 213 × 213 μm. (SPCImage 5.7, https://www.becker-hickl.com).

**Figure 4:**
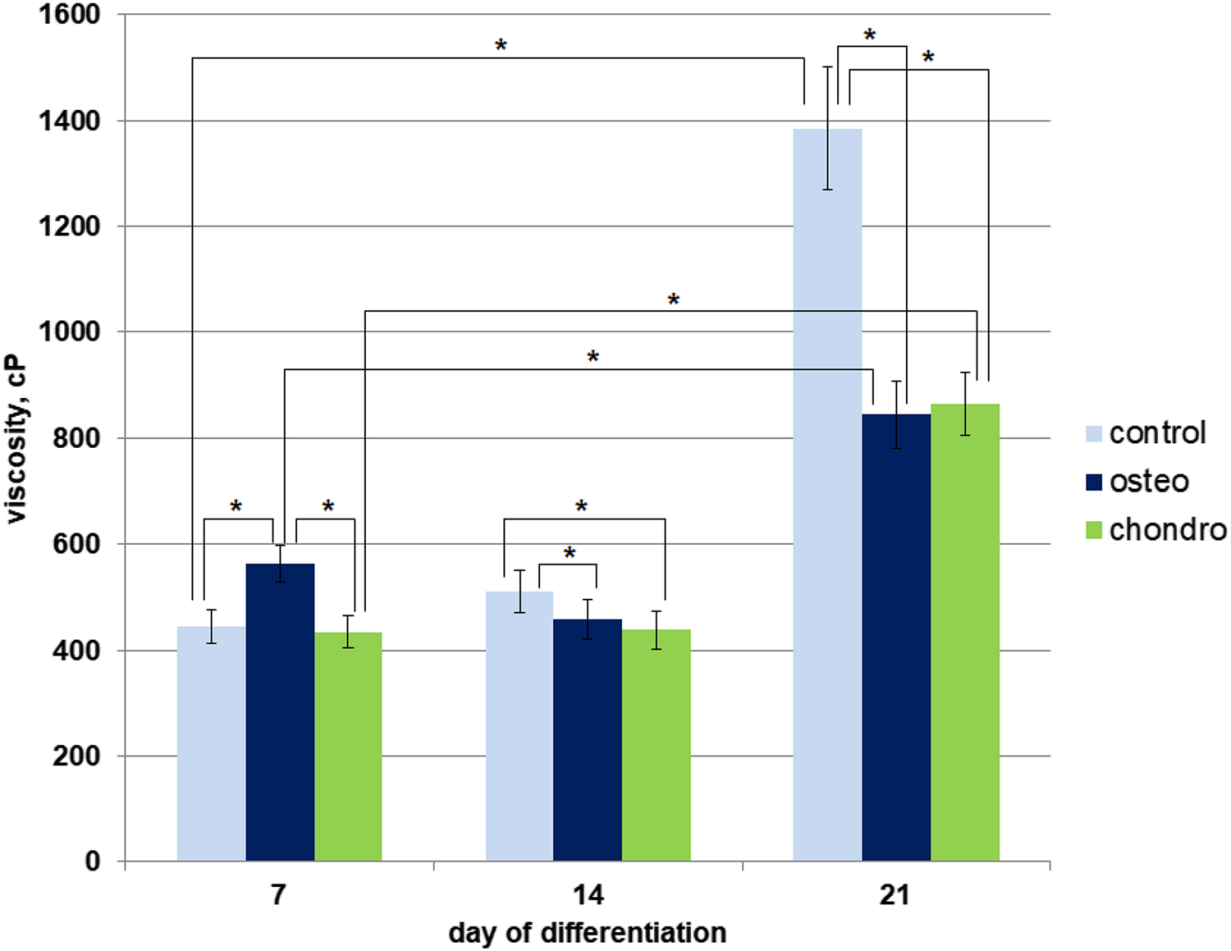
Viscosity analysis of MSCs lipid tail region via FLIM of molecular rotor BODIPY 1 during differentiation. Dynamics of the viscosity change during differentiation. *Statistically significant differences between different conditions with *p* < 0.001.

Having excluded dye aggregation, the biexponential decay kinetics of BODIPY may indicate the fact that the rotor is probing microscopic heterogeneities in the cell membranes. This may be due to the formation of domains of specific viscosity (e.g. lipid rafts, characterized by a higher degree of lipid order), or a vertical distribution of dye positions within a lipid bilayer^10^. Previously, the data recorded from a structurally similar BODIPY dye in porcine eye lens cells were interpreted as two rotor orientations in these membranes, one orientation close to the head region of the bilayer, where the rotor is exposed to a more polar environment and is likely significantly hydrated, and the second component in the highly hydrophobic tail region of the bilayer. It was thought that these two distributions arise due to the liquid ordered (L_o_, lipid raft-like) lipid phase. In the present work we show that in undifferentiated and differentiated MSCs the fluorescence lifetime of BODIPY 1 is indicative of a similar viscosity values as in the lens cells (Figs. 3, 4; SI, Fig. S11). In all examined locations at the plasma membrane BODIPY 1 had a biexponential decay with a short component (τ1) in the region of 500 ps (corresponding to ca 20–30 cP) and a long component (τ2) in the region of 4–5 ns (corresponding to several hundred cP up to 1,500 cP). By analogy with the eye lens membranes^10^ and *E. coli* bacterial cells^13^, we assign the short component of these decays to a BODIPY orientation close to the head region of the bilayer, while the long component corresponds to the viscosity in the inner, highly hydrophobic tail region of the bilayer. The longer component (τ2) was converted to viscosity, as previously discussed. α1 and α2 parameters were used to assess the correctness of the obtained data on fluorescence lifetimes and characterized the contribution of short component (τ1) and long component (τ2), which may reflect the distribution of BODIPY between two locations of the membrane, as previously discussed^10,11^. This is consistent with our experimental study and molecular dynamics simulations of a structurally related BODIPY in a rigid (gel phase) bilayer, where both components were observed and assigned to distinct BODIPY orientations both experimentally and in simulations^11^.

Importantly, in all samples we were able to achieve good plasma membrane staining with BODIPY 1, using our incubation protocol. While some internal organelle staining was observed, it was possible to analyze the plasma membrane by selecting plasma membrane localization from FLIM images by hand. In every point of the image the observed lifetime (longer component τ2) was converted to viscosity using our calibration (SI, Fig. S5). In the case of chondrogenic differentiation, the difference in viscosity of plasma membranes at day 7 was statistically insignificant as compared with control, while in osteogenic differentiating MSCs the viscosity values slightly increased with a statistically significant difference from the control (554.19 ± 33.47 cP vs. 440.57 ± 31.14 cP, respectively). However, a statistically significant decrease in viscosity was observed for both samples at day 14 when compared with control at day 14 (osteo; 458.2 ± 37.02 cP and chondro: 438.04 ± 36.67 cP vs. 510.47 ± 40.27 cP in control). A similar trend was demonstrated on day 21 for both directions of differentiation (osteo: 844.67 ± 63.64 cP and condro: 864.44 ± 59.77 cP vs 1,384.81 ± 116.83 cP in control). Furthermore, our analysis indicates that a statistically significant increase in membrane viscosity was observed on day 21, relative to samples at 7 and 14 days. This was true for undifferentiated MSCs and both types of differentiating MSC.

### Lipid analysis of MSCs membrane during differentiation by ToF–SIMS

It is well known that differentiation of MSCs results in extensive remodeling of the plasma membrane, producing cell-specific membrane compositions and biophysical properties. In simple synthetic model membranes biophysical properties are determined by lipid composition^23,24^. There is evidence from mass spectrometry analysis of lipid composition of differentiated cells^2,4^ and from generalized polarization studies^1^, that differentiation is accompanied by significant changes of membrane lipid composition and order. We hypothesized that MCSs differentiation should be accompanied by changes in membrane lipids, leading to viscosity changes. Therefore, in addition to the FLIM study described above we also performed a detailed analysis of lipid composition from our cell samples by ToF–SIMS.

**Figure 5:**
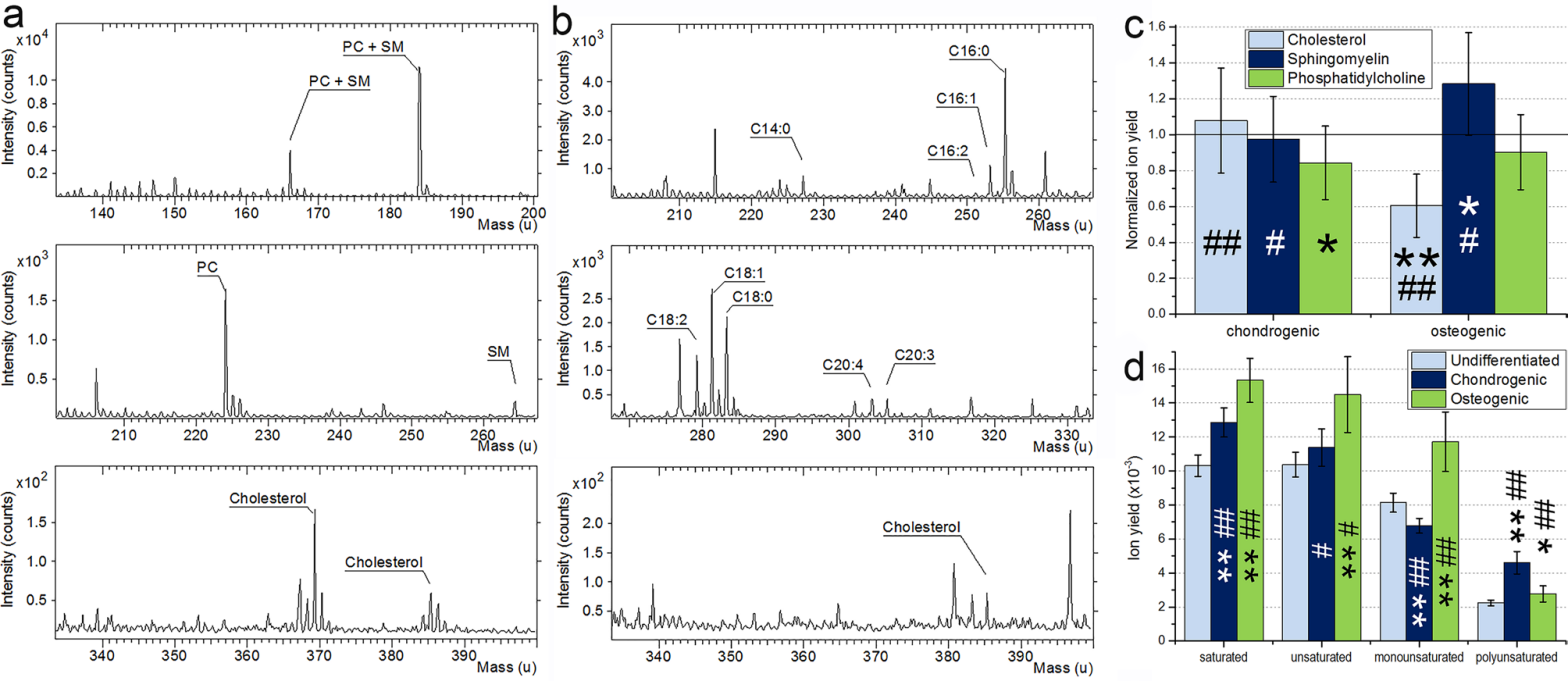
Lipid analysis by ToF–SIMS on day 21. A sample mass spectra of undifferentiated MSCs in (**a**) positive ion mode and (**b**) negative ion mode; (**c**) ion yield for chondrogenic and osteogenic cells, relative to an undifferentiated MSCs (solid line at 1.0). (**d**) fatty acids ion yield for undifferentiated, osteogenic and hondrogenic cells. * and **denotes statistically significant differences with *p* < 0.01 and *p* < 0.001, respectively for chondrogenic/osteogenic differentiating MSCs, compared with control MSCs. # and ## denotes *p* < 0.01 and *p* < 0.001, respectively for chondrogenic compared with osteogenic differentiating MSCs.

In order to test possible correlations of the lipid composition with changes in membrane viscosity, lipid analysis on the 21st day of differentiation was performed by ToF–SIMS, based on comparison of signal intensity of known lipid species^25–27^ (Fig. 5).

The attention was focused on sphingomyelin (SM) and cholesterol signal as these lipids are known to have influence on the membrane viscosity. Figure 5a,b shows a typical mass spectrum of a plasma membrane, only the mass range of analyzed lipid ions is shown. The chemical structure of sphingomyelin is similar to phosphatidylcholine (PC) structure. Thus, most head group ions (m/z 86, 104, 166 and 184) are identical for both lipids. Draude et al. previously showed that SM could be effectively distinguished from PC by m/z 264 and 224 ions, respectively^27^. To evaluate cholesterol signal ion with m/z 369 was used. Figure 5c shows ion yields of main lipid species (cholesterol, sphingomyelin, phosphatidylcholine) relative to the control (undifferentiated 21 day cells) sample yields, line 1.0. It could be seen that the cholesterol yield for chondrogenically differentiated cells increases, while in osteogenically differentiated cells it significantly decreases (by 40%), compared to undifferentiated 21 day MSCs. The trend for sphingomyelin ion is reversed; a decrease for chondrogenically differentiated cells and a significant increase (by 23%) for osteogenically differentiated cells was observed, compared to undifferentiated 21 day MSCs. Phosphatidylcholine yield slightly decreased in both cases of MSCs differentiation, despite the identical density of cellular monolayers.

Both cholesterol and spingomielin are likely to contribute to a change in viscosity of cells following differentiation. The effect of cholesterol depends on the nature of the initial lipid phase. In gel phases, an addition of cholesterol to the model bilayer can cause a significant decrease in the viscosity of large unilamellar vesicles made of EYSM (egg yolk sphingomyelin), according to a molecular rotor study^11^. Wu et al. also revealed that in the case of the sphingomyelin rich model membrane the addition of up to 40% cholesterol contributed to 10% decrease in viscosity, probably due to a gradual change of the gel phase into the liquid ordered (L_o_) phase^21^.

Polyunsaturated fatty acids (PUFAs) and their bioactive derivatives affect the proliferation and differentiation of various stem cells^28^. Therefore, we directed our attention to the unsaturated/saturated fatty acid ratio, Fig. 5d. FA are likely originated from phospholipids and acylglycerols as a fragment ions. Nine main species were clearly detected including saturated acids (C14:0, C16:0, C18:0), monounsaturated acids (C16:1, C18:1) and polyunsaturated acids (C16:2, C18:2, C20:3, C20:4). Other FA chains may also be present in the spectra but the evaluated was omitted due to a low signal to noise ratio.

Figure 5d shows saturated and unsaturated fatty acids ion yield for control, osteogenically and chondrogenically differentiated MSCs. While unsaturated/saturated fatty acid ratio for all three cell types remains within the statistical error it could be clearly seen that fatty acids ion yield increases for differential MSCs compare to undifferentiated cells. It likely indicates that overall lipid content increases for differential cells. Interestingly chondrogenic MSCs shows increase for polyunsaturated fatty acids and decline for monounsaturated fatty acids. It means that despite similar saturated and unsaturated fatty acids ratio, lipids in chondrogenically differentiated MSCs plasma membranes contain a higher percentage of polyunsaturated chains and lower percentage of monounsaturated chains.

## Discussion

In this study, we investigated the membrane viscosity changes in stem cells during differentiation on the basis of fluorescence lifetimes of a new viscosity sensitive molecular rotor BODIPY 1, with good aqueous solubility. Firstly, we ascertained that the lifetime of the BODIPY rotor uniquely responds to the viscosity of its lipid environment (i.e. lipid packing) and is not affected by the dye aggregation. While stem cell membranes are likely to be complex, containing significant number of proteins and their complexes, we are confident that our molecular rotor specifically probes the lipid packing, (rather than binding to proteins), based on our previous method validation. Specifically, (1) our previous studies of model lipid membranes that compared FLIM with molecular rotors to molecular dynamic simulations, and fluorescence correlation spectroscopy (FCS) and anisotropy data^11,29^ indicated that the rotor-derived viscosities are similar to those obtained using the above methods. (2) Photochemical quenching data for BODIPY rotors imbedded within membranes indicated that the biexponential decay kinetics originated from two vertically offset subpopulations of the rotor^22^. (3) Previous studies of highly packed membranes of live cells, assigned to L_o_ or lipid raft phases, give biexponential decays and show close similarities (in decay profiles and in temperature responses) to L_o_ model membranes of known composition^10,13^. This data allow us to assign BODIPY rotor lifetimes, observed in MSC cells before and after differentiation, to lipid bilayer viscosity, with high degree of confidence.

Based on the FLIM data, we registered a large increase in membrane viscosity in all cells on day 21 of culturing, compared to day 0 values (SI, Fig. S13). At the same time, osteogenically and chondrogenically differentiated cells were characterized by a lower viscosity on day 21, compared to a undifferentiated 21 day cells. The detailed lipid analysis by ToF–SIMS revealed the cholesterol level increase in chondrogenically differentiated cells and sphingomyelin level increase in osteogenically differentiated cells, compared to control cells at day 21. Unfortunately, we were not able to perform the accurate mass spectrometry analysis of the lipid composition in undifferentiated cells on day 0 of cell cultivation (characterized by a low membrane viscosity), due to a vastly different cell density (data in SI, Figs. S14, S15).

An increasing body of evidence has recently appeared suggesting that biophysical and biochemical properties of plasma membranes are clearly specific for each cell type. Mass spectrometry data provided evidence that lipidome remodeling occurs during stem cell differentiation; the new phenotype of remodeled membranes may reflect the specific functioning of the resulting cell types including secretion of extracellular matrix components^2^. Thus, the characterization of membrane viscosity or fluidity in various lineages is desirable in order to link membrane properties with cellular identity upon differentiation.

Taking into account the wide application of mesenchymal stem cells in cell therapy and tissue engineering, the study of viscosity in cells, derived from MSCs deserves special attention. It should be noted that published data on the viscosity properties in stem cells during differentiation are very scarce. Tan et al. used micropipette aspiration technique and detected that human adult bone marrow-derived mesenchymal stem cells behave like a viscoelastic solid material, consistent with other cell types such as chondrocytes and endothelial cells^30^.

Daniels et al. used multiple particle tracking microrheology to show that human induced pluripotent stem cells have a more viscous phenotype that parental fibroblasts^31^. Matsuzaki et al. unveiled the membrane fluidity landscapes in various lineages ranging from human pluripotency to differentiated progeny using fluorescence-based generalized polarization method^1^. Other works provide only an indirect understanding of the viscosity properties in differentiating stem cells based on the lipid analysis^2,4^. Based on the above, it is clear that to date, membrane viscosity monitoring in mesenchymal stem cells during differentiation has not been carried out.

Here we reported the direct recording of the mesenchymal stem cells membrane viscosity during osteogenic and chondrogenic differentiation. This work used the viscosity-dependent lifetimes of a new molecular rotor BODIPY 1, which allowed viscosity imaging with excellent spatial resolution using FLIM.

Using BODIPY 1 we detected that in the case of both chondrogenic and osteogenic differentiation the viscosity values increased on day 21, compared to day 0 (SI data, Fig. S13). Only small statistically insignificant variations in viscosity were detected on day 7 and 14 for both types of differentiation. We note that undifferentiated cells on day 21 were also characterized by surprisingly high membrane viscosity values, Fig. 4. We are unsure of the origins of this high viscosity but hypothesize that this was caused by an extremely high cell density in these samples (the cells densities between undifferentiated and differentiated cells were nearly identical, as was revealed by light microscopy). We performed control staining for both types of differentiation, given the high cell density could have resulted in spontaneous differentiation, however, both types of staining gave negative results in these samples. We measured the viscosities of the plasma membrane in excess of 1,000 cP in some cases of differentiation. The lipid tail region viscosity represents the value that affects the diffusion of small molecules as well as macromolecules in the bilayer. The lipid bilayer viscosity affects diffusion-controlled processes, which may include the activity of proteins, including receptor and transport proteins, which are involved in the processes of cell adhesion, migration and proliferation. In the study^1^ authors using generalized polarization showed that the stem cells plasma membrane becomes more rigid during differentiation due to more ordered membranes. We also suggest that the more viscous membranes of differentiated MSC can become more rigid.

Mass spectrometry was performed for control cells as well as chondrogenically and osteogenically differentiated cells on day 21. The cholesterol ion yield of chondrogenically differentiated cells increased against the background of other ions, while sphingomyelin level decreased. It is well known that the products of SM metabolism, such as ceramides, sphingosine, S1P and DAGs, function as cellular signaling molecules, participating in cell growth, cell differentiation and programmed cell death^32^. We suggest that during chondrogenic differentiation MSCs mobilize the SMs to produce the secondary metabolites necessary for differentiation towards chondrocytes, which leads to a decrease in the level of SMs.

In addition, we showed the PC level decrease. The phosphocholine represents choline storage in vivo and its quantity is related to the size of the cell membrane. Although there are conflicting results regarding PC and membrane size, we hypothesize that PC decrease may be connected with the size of the cell membrane So Jang et al. showed that phosphocholine represents choline storage in vivo and is related to the size of the cell membrane. The authors showed phosphocholine increase during chondrogenic differentiation, although cell proliferation was not detected by either DNA assays or cell counting in the present study. Thus, the increased phosphocholine level during chondrogenesis was likely caused by MSC differentiation into chondrocytes rather than by proliferation. The authors infer that chondrocytes are bulkier than MSCs, as the phosphocholine level relates to the size of the cell membrane, which covers the entire cell, and is higher in chondrocytes than in MSCs that do not proliferate^33^. However Beatriz Rocha et al. using MALDI MSI and TOF SIMS reported the decrease of PC levels on day 14 of chondrogenic differentiation, which was related to a smaller size of chondrogenically differentiated MSCs. They showed that after 14 days of chondrogenesis, the resulting chondrocyte differentiating MSCs are smaller in size than noninduced MSCs because during chondrogenesis BMSCs stop proliferating to begin differentiation into chondrocytes^4^. It should be noted that in our present work we examined lipid composition only for cells on day 21 of differentiation, at later stages of chondrogenic differentiation of MSCs compared to the study in^4^.

Moreover, we detected that the monounsaturated fatty acids signal of chondrogenically differentiated MSCs is lower than that of undifferentiated cells. On the contrary, polyunsaturated fatty acids signal level of chondrogenicaly differentiated MSCs increased relative to osteogenically differentiated MSCs and undifferentiated cells. Based on our present data and the data of Levental et al.^2^ we suggest that PUFA elevation may be associated with the chondrocyte membrane features.

Osteogenically differentiated cells on day 21 showed a viscosity decrease relative to undifferentiated 21 day cells, similarly to chondrogenically differentiated cells. However, as revealed by the mass spectrometry analysis, the reasons for this viscosity decrease are completely different. The cholesterol ion yield of osteogenically differentiated cells significantly decreased (by 40%) against the background of other ions, while sphingomyelin levels increased. Previously, Levental et al. showed that osteoblasts had a low level of cholesterol^2^, consistent with our data. It is well known that sphingomyelin is necessary for bone and dentin mineralization. Therefore, the increased levels of sphingomyelin in osteogenicaly differentiated cells may be necessary for its consumption for ECM mineralization^34^. Potentially, a decrease in phosphatidylcholine ion yield may also be related to a smaller size of osteogenic differentiated MSCs as in the case chondrogenic differentiation. Levental et al. showed that the plasmalogen forms of PE and PC were significantly increased in osteoblasts relative to undifferentiated MSCs. However, these authors observed an increase in PC at an earlier differentiating date (14 days). We also detected an increased signal level of polyunsaturated fatty acids in osteoblast when compared to undifferentiated MSCs. Likewise, in^2^ both the lipid length and the unsaturation changes were highly significant (with 34% more long lipids (≥ 36 carbon acyl chains) and 61% more polyunsaturated lipids) in osteoblasts compared to undifferentiated MSCs.

Thus, it appears from our data, that, while both types of differentiation induce a decrease in membrane viscosity, the molecular mechanism of this decrease is different for each type of differentiation. The change in the lipidome of the differentiated cells likely reflects their specific functions following differentiation.

It is known, that stem cell maintenance and differentiation depends on membrane fluidity, possibly through the modulation of intracellular signaling. Matsuzaki et al. hypothesized that acceleration of differentiation may be associated with the transfer of membrane stiffness to neighboring cells^1^. Salaita et al. emphasized that intermembrane signaling is initially triggered by the clustering of adhesion ligands in the fluid membrane^35^. Such physical connections among cells with different fluidic membrane potentials can strengthen cell–cell signaling, leading to the ‘‘relay’’ of membrane fluidity signatures. Our results directly demonstrate for the first time that differentiation of MSCs results in distinct membrane viscosities, that reflect the change in lipidome of the cells following differentiation.

## Materials and methods

### Stem cell culture, osteogenic and chondrogenic differentiation

All investigations involving human cells were performed according to the Tenets of the Declaration of Helsinki and were approved by Ethics Committee of Privolzhsky Research Medical University, Nizhny Novgorod. Signed informed consent was obtained from donors. Biopsy samples of the fat tissues were processed within 8 h after abdominal liposuction. Stromal cells of the fat tissue were isolated using Zuk’s method^19^ with modifications. The tissue was washed in Hank’s saline solution with gentamicin (200 units/ml), cut with scissors and incubated with a 0.1% solution of collagenase type I (Worthington, USA) at 37 °C for 90 min with continuous mixing. The enzyme was then inhibited with 10% calf serum (BioloT, Russia). Mature adipocytes were isolated by centrifuging at 300 g for 10 min. The pellet of cells was washed free of the enzyme in DMEM medium (Sigma, Germany) containing 10% fetal bovine serum (FBS) (GE Healthcare Hyclone, Logan, UT, USA). The cell suspension was filtered through a nylon filter (pore diameter 100 µm) and centrifuged in a density gradient (Histopaque-1077; Sigma, Germany) at 400 g for 30 min at ambient temperature to obtain fractions of mononuclear cells. The cell suspension was washed from Histopaque-1077 in DMEM medium three times. The MSCs were cultured in MesenCult MSC Basal Medium (Human) (Stemcell Technologies, Vancouver, BC, Canada) supplemented with 10% FBS, 0.58 mg/ml l-glutamine (PanEco, Moscow, Russia) and 40 U/ml gentamicin. The medium was changed every 3–4 days. The cell culture was maintained at 37 °C in a 5% CO_2_ humidified atmosphere.

The MSCs were tested using a flow cytometer (FACSAria III; BD Biosciences, USA) for the presence of typical markers of MSCs (CD105, CD73, CD90, CD34, CD45, HLA-DR, CD11b, and CD19)^36^. The commercial Human MSC Analysis Kit (BD Biosciences, USA) was used for characterization.

Differentiation was induced by incubating the MSCs in MesenCult Osteogenic Stimulatory Kit (Human) (Stemcell Technologies) or Stem MACS Chondro Diff Media (MASC, Miltenyi Biotec GmbH, Bergisch Gladbach, Germany). The medium was replaced every 3–4 days during 3 weeks of the experiment.

Morphological changes were assessed by counting the number of cells with fusiform and polygonal morphology at each differentiation stage. Osteogenic and chondrogenic differentiations were verified by staining of the calcification of the extracellular matrix with Alizarin Red S (Sigma-Aldrich, St. Louis, MO, USA,) and acidic polysaccharides such as glycosaminoglycans in cartilage with Alcian blue (Sigma-Aldrich), respectively^37^.

For microscopic imaging, 5 × 10^5^ cells were transferred into a sterile dish with a cover glass bottom (0.17-mm thick) and incubated for 24 h until cells attachment to the glass surface. Cell monolayers were used. Cells were imaged before the induction of differentiation (day 0) and on days 7, 14, and 21 of osteogenic and chondrogenic differentiation. Untreated cells served as controls for all time points^36^.

### BODIPY synthesis

The chemical structure of the dyes used in this study is shown in Scheme 1. The synthesis of BODIPY 2 was described previously^12^. The new molecular rotor BODIPY 1 was synthesized by modification of the above published procedure^12,20^. Full synthetic details and characterization of intermediates and main product can be found in the supplementary information file (SI, Scheme S1; Fig.S1–S3).

Viscosity calibration of the fluorescence lifetime of BODIPY 1

A mode-locked femtosecond Ti:Sapphire laser (Chameleon Vision II, Coherent Inc., Germany) tunable over the 680–1,080 nm range (140 fs pulse duration, 80 MHz) was used as an excitation source. The excitation light was frequency doubled using a second harmonic generation (SHG) crystal (Harmonic, Coherent Inc., Germany). The excitation was performed at 480 nm and fluorescence was detected at 520 nm after passing through a 500 nm long-pass filter. The detection system consisted of a DCC-100 detector control module (Becker&Hickl, Germany), PMC-100–1 photomultiplier tube (Hamamatsu, Japan), Omni-λ 150 monochromator (LOT-Quantum Design, Germany), and SPC-830 single-photon counting card (Becker&Hickl, Germany). The measurements were performed on 1 μM solutions of BODIPY 1 in quartz cuvettes with a 1 cm path length, measured in a qpod cuvette holder heated using a TC 125 Peltier thermostat (Quantum Northwest, USA). Measurements were performed in a temperature range of 1–100 °C. The temperature was checked before and after each decay acquisition with a thermocouple. The number of photons at the peak of all traces was 10, 000 counts. The viscosity values for glycerol solutions at various temperatures were taken from^38^.

### Cell staining with BODIPY dyes

The control cells (undifferentiated MSCs) and differentiated MSCs were stained with BODIPYs 1 or 2 according to the published procedure^12^ with modification. Before staining, the medium was replaced with cold Hank's solution without magnesium or calcium ions and phenol red, in order to induce the selective staining of the plasma membrane and to slow down endocytosis. The Hanks solution was then replaced with a cold PBS solution containing 26.7 μM of BODIPY, which was diluted from 1 mM DMSO stock (final concentration of DMSO 1 × 10^−4^%). Due to the formation of a multilayer cell structure as a result of the differentiation process, it was necessary to increase the rotor concentration to 26.7 μM (× 3 compared to the previously used in^10^ and to incubate with gentle shaking for 30 min at + 4 °C. The incubation solution of the dye was not washed for imaging. Imaging was conducted at + 18 °C. The staining of MSCs with a previously published probe BODIPY 2^16,17^ was not successful, due to its poor uptake and brightness in the MSCs culture.

### Multiphoton fluorescence microscopy and FLIM

The fluorescence intensity and FLIM images of BODIPYs 1 and 2 were obtained using an LSM 880 (Carl Zeiss, Oberkochen, Germany) inverted laser scanning confocal microscope equipped with a time-correlated single photon counting (TCSPC) system (Sim-ple-Tau 152, Becker and Hickl GmbH, Berlin, Germany). BODIPY fluorescence was excited with a Chameleon Vision II (Coherent, Santa Clara, CA, USA) Ti:Sa femtosecond laser, using an 80 MHz repetition rate and a pulse duration of 140 fs at wavelengths of 850 nm. Emission was detected in the ranges 500–550 nm for monomers, and 598–660 nm for aggregates of BODIPY. 8,000–10,000 photons was collected per decay curve. The constant photon count rate during image acquisition lead to the absence of photobleaching. The average power of the Ti:Sa laser was measured using a PM100A power meter (ThorLabs Inc., Newton, NJ, USA). An internal microscope reference, reporting the two-photon excitation efficiency, was used to control the laser power in all experiments. The average power incident on the samples was approximately 6 mW. A C-Apochromat 40 × /1.2 water immersion objective was used for image acquisition^37^.

### Fluorescence lifetime analysis

FLIM images of 256 × 256 pixels and 512 × 512 pixels were obtained. FLIM data were analysed in the SPCImage software (Becker&Hickl, Germany) using a biexponential decay model (incomplete multiexponential). To maintain a minimum peak count in the decay of 1,000 counts per pixel we used binning 2. The scatter parameter was fixed across the image to account for scattered light from a thick cell layer. A pseudocolour scale was assigned to each fluorescence lifetime, amplitude and the goodness of fit χ^2^ values (red for small values and blue for large values) to provide the corresponding lifetime maps. In these experiments, fluorescent lifetime of the long decay component was used for the viscosity analysis. The viscosity values we determined according to the calibration of BODIPY 1 in methanol/glycerol solutions of known viscosity (SI, Figs. S4–5).

### Sample preparation for ToF–SIMS

For analisis 5 × 10^5^ MSC were tranfered on plates, which were coated with a polylysine layer to provide additional cell adhesion to the substrate. Thereafter some plates were placed in MesenCult MSC Basal Medium (Human) (Stemcell Technologies, Vancouver, BC, Canada) supplemented with 10% FBS, 0.58 mg/ml l-glutamine (PanEco, Moscow, Russia) and 40 U/ml gentamicin, other plates were placed in differentiating medium: MesenCult Osteogenic Stimulatory Kit (Human) (Stemcell Technologies) and Stem MACS Chondro Diff Media (MASC, Miltenyi Biotec GmbH, Bergisch Gladbach, Germany). The medium was changed every 3–4 days. The cell culture was maintained at 37 °C in a 5% CO_2_ humidified atmosphere^37^. 3 week later cells were fixed in 4% PFA solution. The surface of glasses with adsorbed cells was washed three times with PBS. Then, the samples were washed once with Milli-Q water to remove excess salts. Since ToF–SIMS analysis involves studies under vacuum condition, a cell dehydration was applied. Drying was carried out under gentle argon stream. In this process, the nozzle is positioned so that the argon blows the liquid across the surface of the substrate as previously described by Berman et al.^39^. Drying was carried out under argon condition.

### ToF–SIMS analysis

Mass spectrometry measurements were performed on a ToF–SIMS 5 instrument (ION-TOF Gmbh, Germany) equipped with a 30 keV Bi_3_^+^ liquid metal ion source. 18 mass spectra were recorded for each sample containing confluent layers of cells in spectroscopy mode for both positive and negative secondary ions. A randomly selected area of 300 × 300  μm^2^ with a resolution of 64 × 64 pixels was scanned for every mass spectrum. The primary ion current, measured by a Faraday cup, was 0.4 pA corresponding to the primary ion dose density ~ 4 × 1,011 ions/cm^2^, hich was below static SIMS limit. A low-energy electron flood gun was activated to avoid charging effects. Ion yields were calculated as an intensity of the corresponding peak of interest normalized to the total ion count amount^26^.

### Statistical analysis

Due to a significant internalization of BODIPY 1 it was impossible to analyze lifetime histograms from the whole field of view and, hence, the decays for analysis were selected point by point, ensuring the membrane location of the pixels selected. For each time point of the differentiation activity, from 23 to 78 randomly selected cells and from 47 to 120 pixel decays were inspected. The statistical analysis was performed using STATISTICA 64 software, version 10 (StatSoft Inc., Tulsa, OK, USA). Mean and standard deviation (SD) values were used to express the data. Differences in the mean values were tested for significance using the Student’s *t* test or the one-way ANOVA with Fisher’s post hoc test (*p* ≤ 0.05).

## Supplementary information

Supplementary Information.
