# Supplementary material for: Monitoring membrane viscosity in differentiating stem cells using BODIPY-based molecular rotors and FLIM
Source: Sci Rep. 2020 Aug 20;10:14063. doi: 10.1038/s41598-020-70972-5 (PMC7441180; doi:10.1038/s41598-020-70972-5)
Supplement: Supplementary file 1 — Supplementary Information. [file 41598_2020_70972_MOESM1_ESM.pdf]

Monitoring membrane viscosity in differentiating stem cells using BODIPY-based molecular rotors and FLIM

Alena S. Kashirina[1], Ismael López-Duarte[2], Markéta Kubánková[2], Alexander A. Gulin[3,4], Varvara V. Dudenkova[1], Svetlana A. Rodimova[1,5], Hayk G. Torgomyan[1], Elena V. Zagaynova[1,5], Aleksandra V. Meleshina[1*], Marina K. Kuimova[2*]

Supplementary Material

1. Synthesis and compound characterisation

1.1 General Materials and Methods

The manipulation of all air and/or water sensitive compounds was carried out using standard inert atmosphere techniques. All chemicals were used as received from commercial sources without further purification. Anhydrous solvents were used as received from commercial sources. Analytical thin layer chromatography (TLC) was carried out on Merck aluminium backed silica gel 60 GF254 plates and visualisation when required was achieved using UV light or I_2. Flash column chromatography was performed on silica gel 60 GF254 using a positive pressure of nitrogen with the indicated solvent system. Where mixtures of solvents were used, ratios are reported by volume. Nuclear magnetic resonance spectra were recorded on 400 MHz spectrometers at ambient probe temperature. Chemical shifts for ^{1}H NMR spectra are recorded in parts per million from tetramethylsilane with the solvent resonance as the internal standard (methanol: δ = 3.31 ppm). ^{13}C NMR spectra were recorded with complete proton decoupling. Chemical shifts are reported in parts per million from tetramethylsilane with the solvent resonance as the internal standard ($^{13}CD_3OD$: 49.00 ppm). ^{19}F NMR spectra were recorded with complete proton decoupling. Chemical shifts are reported in parts per million referenced to the standard hexafluorobenzene: −164.9 ppm. Mass spectra were carried out using ElectroSpray Ionisation (ESI), and only molecular ions are reported.

1.2 Synthetic Procedures

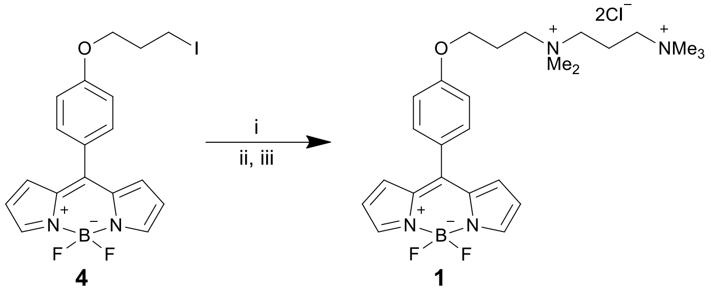

Scheme S1. Synthesis of BODIPY 1: (i) *N,N,N',N'*-tetramethyl-1,3-propanediamine, THF, RT and then (ii) CH_3I, DMF, RT and finally (iii) Dowex 1x8 200 mesh ion-exchange column, H_2O. Yield: 41%. BODIPY 4 was prepared following a reported procedure.[1]

BODIPY 1. To a solution of BODIPY 4 (220 mg, 0.48 mmol) in 16 mL of dry THF was added *N*,*N*,*N'*,*N'*-tetramethyl-1,3-propanediamine (3 mL, 17.9 mmol) under N_2 atmosphere. The reaction mixture was stirred at 18 ^{0}C for 24 h. The resulting dark-red precipitate was filtered, washed with THF (50 mL) and diethyl ether (100 mL) and dried under vacuum to give the corresponding mono-charged intermediate which was used without further purification. Then, the mono-charged intermediate was dissolved in DMF (3 mL) and iodomethane (1 mL, 6.5 mmol) was added to the solution under N_2 atmosphere. The reaction mixture was stirred overnight at room temperature, and then the solvent was evaporated under reduced pressure to give a dark red crude product which was purified by column chromatography on silica gel (methanol, and then a mixture of 3:1 methanol:0.5M NH_4Cl), R_f 0.2. Fractions were evaporated at 30°C to give a mixture of BODIPY 1 and NH_4Cl which was further dissolved in methanol and successively filtered to remove most of NH_4Cl. The red-orange crude, which was still contaminated with NH_4Cl according to ^{1}H-NMR, was dissolved in methanol and a saturated solution of NH_4PF_6 in H_2O was added in order to exchange counter-ions. The precipitate was isolated by filtration, washed thoroughly with H_2O, methanol and diethyl ether. Finally, the red-orange solid was dissolved in acetone and passed through a Dowex 1x8 200 mesh ion-exchange column (H_2O). Fractions were evaporated to dryness (at 30°C) to give BODIPY 1 as a red-orange wax. Yield: 108 mg (41%).

^{1}H NMR (400 MHz, CD_3OD) δ_H 7.92 (br s, 2H), 7.62 (d, *J* = 8.7 Hz, 2H), 7.22 (d, *J* = 8.7 Hz, 2H), 7.04 (d, *J* = 4.0 Hz, 2H), 6.64 (dd, *J* = 4.0, 1.4 Hz, 2H), 4.29 (t, *J* = 5.5 Hz, 2H), 3.74 (m, 2H), 3.56 (m, 4H), 3.30 (s, 6H), 3.28 (s, 9H), 2.44 (br, 4H); ^{13}C NMR (100 MHz, CD_3OD) δ_C 162.62, 148.75, 144.71, 136.01, 133.75, 132.58, 127.81, 119.61, 115.95, 66.13, 63.85, 63.73, 61.72, 54.11, 51.75, 24.01, 18.85; ^{19}F NMR (377.5 MHz, CD_3OD) δ_F –145.64 (q, J_{FB} = 28.6 Hz); HRMS (ESI-TOF) m/2z 234.6534 ($C_{26}H_{37}BF_2N_4O$, $[M-2Cl]^{2+}$, requires 234.6526).

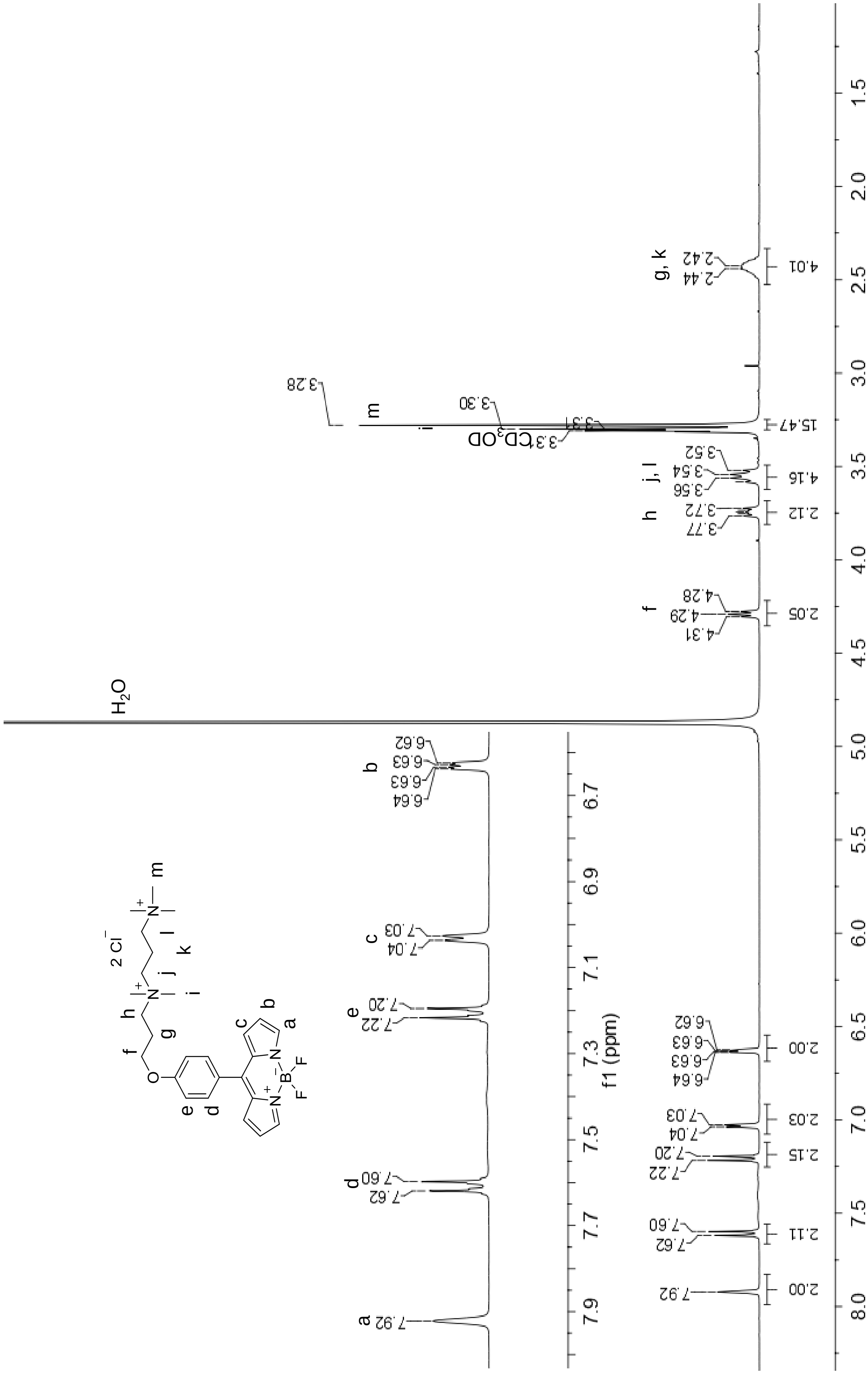

Figure S1. ^{1}H NMR spectrum of BODIPY **1** (400 MHz, CD_3OD).

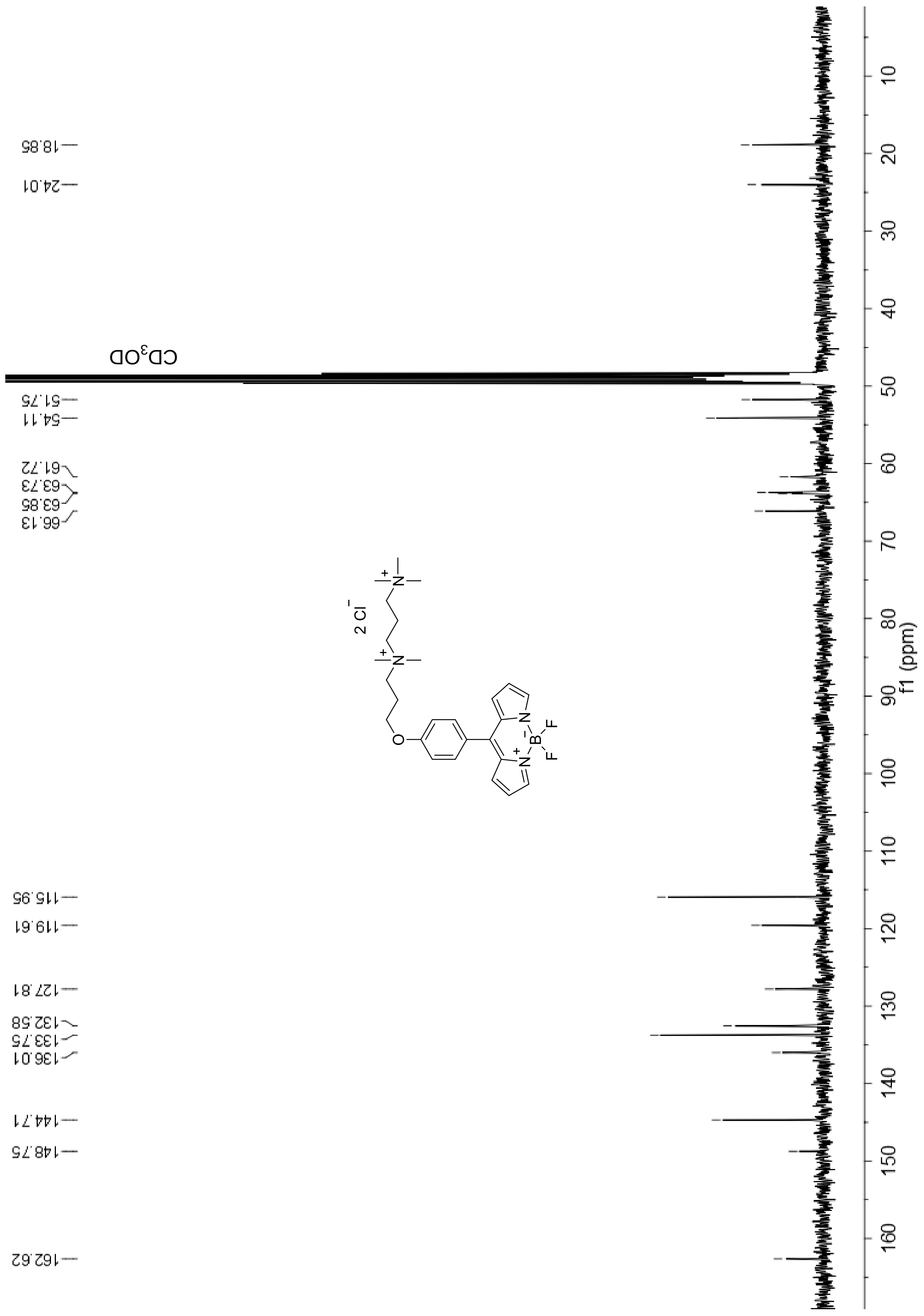

Figure S2. ^{13}C NMR spectrum of BODIPY **1** (100 MHz, CD_3OD).

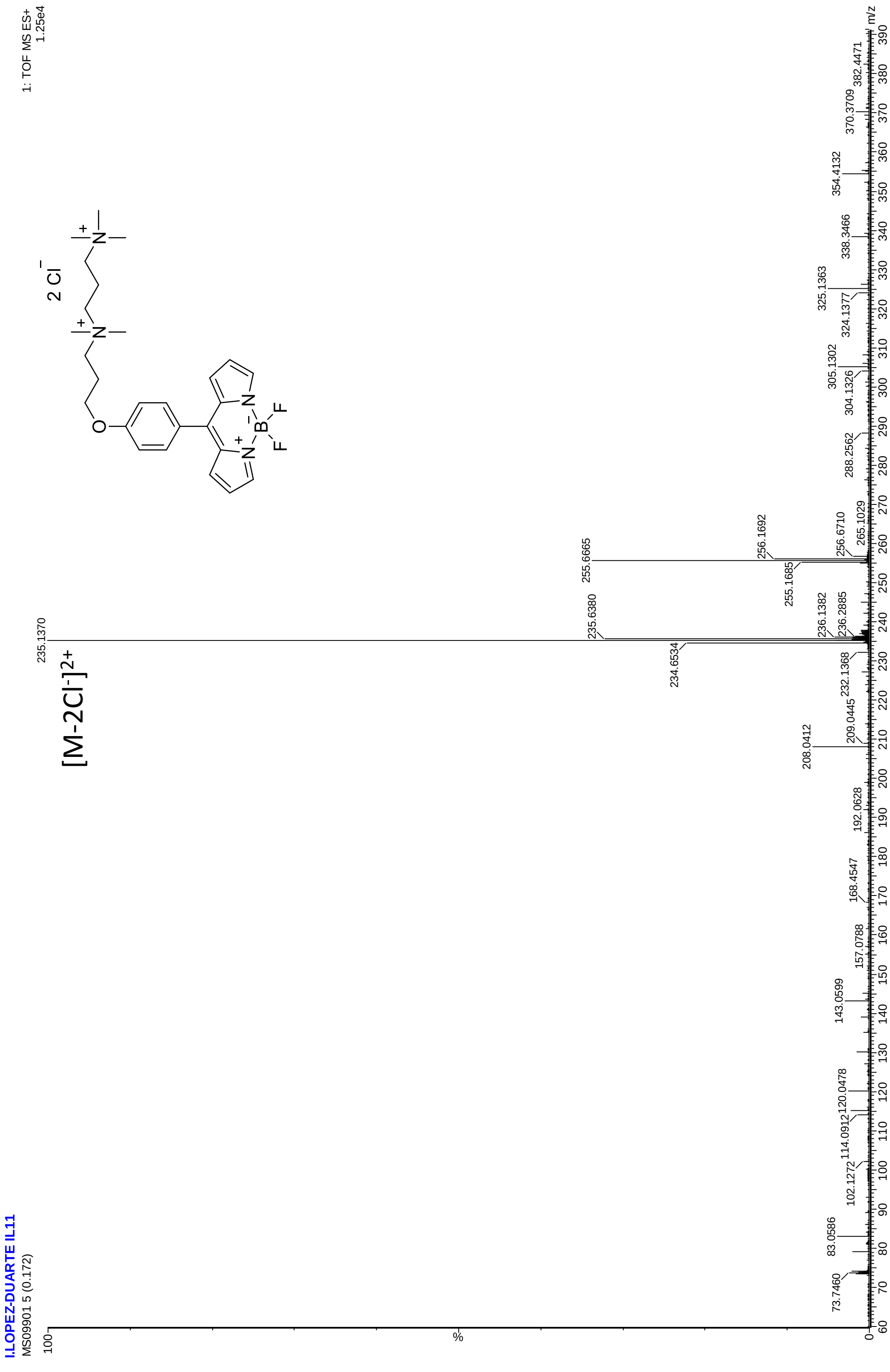

Figure S3. HR MS (ESI+TOF) mass spectrum of BODIPY 1

2. Spectroscopic characterization

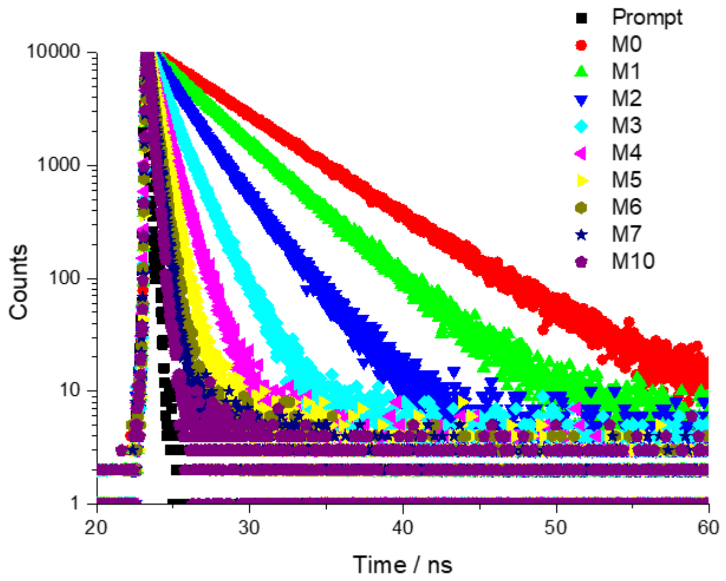

Figure S4. Fluorescence time resolved traces of BODIPY 1 recorded as a function of the decreasing viscosity of the environment (M0→M10), obtained in glycerol at 1.2-100 ^{0}C. The decays were fitted using a monoexponential decay function and the obtained lifetimes were plotted against viscosity in Figure S5.

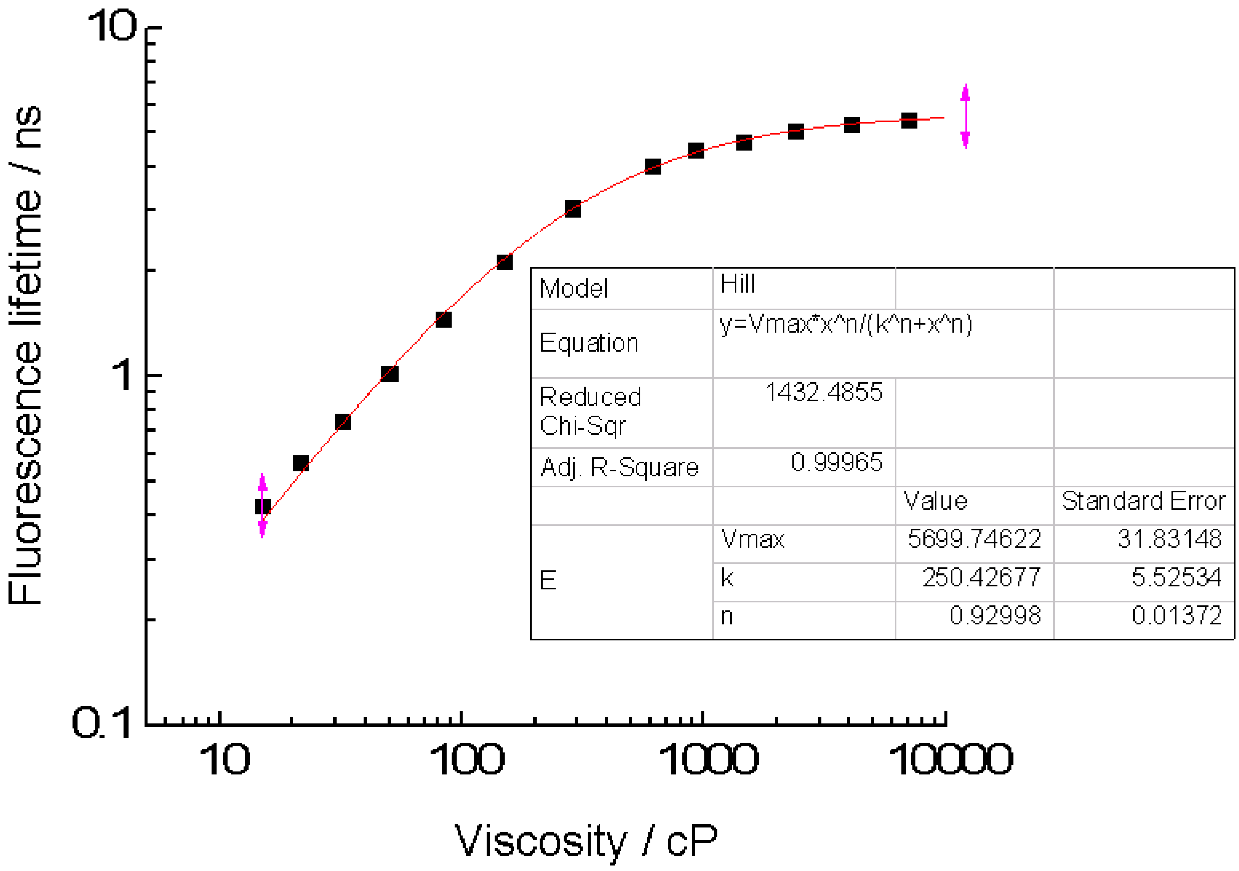

Figure S5. The fluorescence lifetime *vs* viscosity calibration for BODIPY 1. The viscosity dependence of the fluorescence lifetime was fitted by a variant of the Hill function using the following equation:

$$\mathbf{y=Vmax*x^\wedge n/(k^\wedge n+x^\wedge n)} \quad (1)$$

where x is viscosity in cP and **y** is the fluorescence lifetime of the rotor. The fit parameters are given as an insert to Figure S5

3. Imaging experiments

1. Differentiation staining of MSC

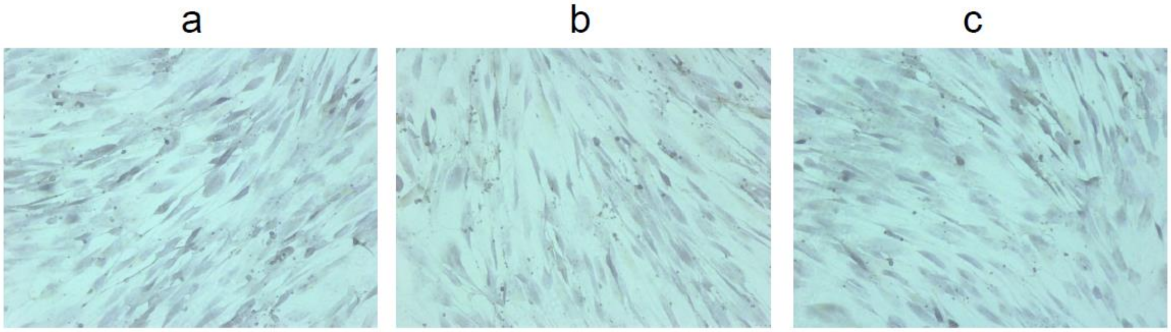

Figure S6. Microscopic images of MSCs in transmitted light. Hematoxylin and Eosin staining of undifferentiated cells on 21 day (a); negative Alizarin Red S staining of undifferentiated cells on 21 day (b); negative Alcian blue staning of undifferentiated cells on 21 day (c). The image size is 1289 × 964 μm.

2. BODIPY 2 staining

Initially we stained control and differentiated MSCs with molecular rotor BODIPY 2, that was previously successfully used in 2D and 3D cell culture and *in vivo* (2, 3). We observed the poor cell staining for this rotor. Higher concentrations of BODIPY 2 in the staining medium resulted in brighter images, however, it was clear from the decay analysis that the lifetimes obtained from cellular membranes change significantly depending on the incubation concentration, which is a signature of dye aggregation.

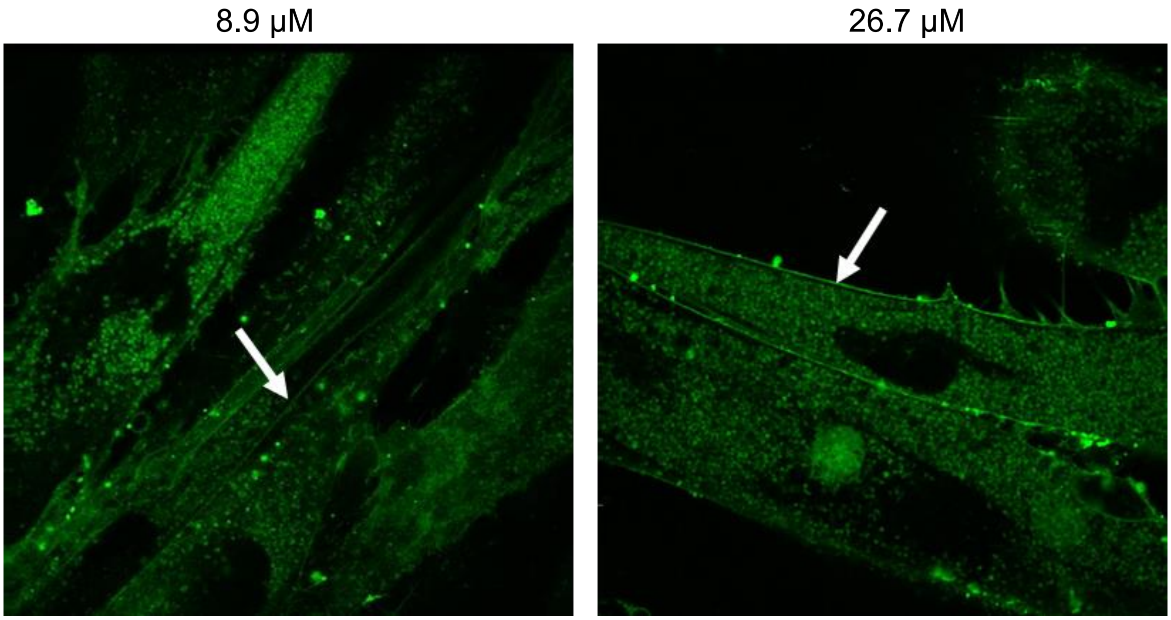

Figure S7. Fluorescent images of MSCs, incubated in the presence of different concentration of BODIPY 2. An example an MSCs plasma membrane staining is shown by arrows. The image size is 213 × 213 μm (1024 × 1024 pixels).

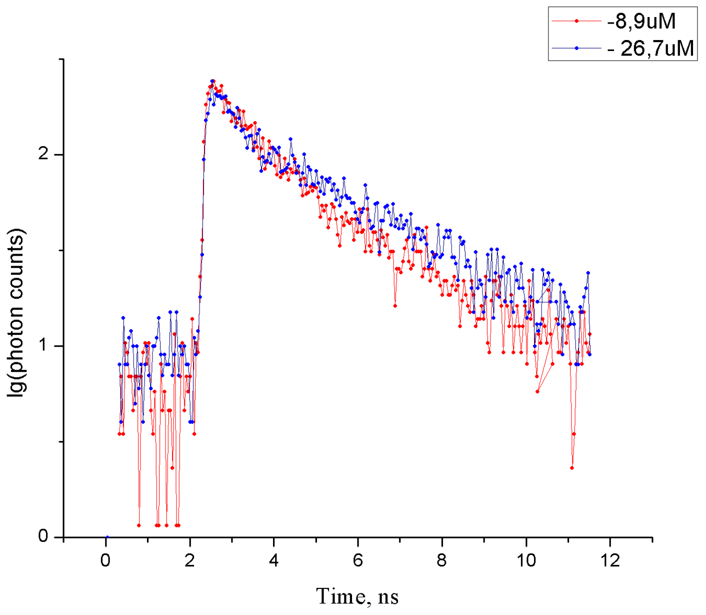

Figure S8. A time resolved fluorescence trace recorded from a plasma membrane location of MSC cells stained with BODIPY 2 at different concentrations: 8.9 μM and 26.7 μM respectively. Since the shape of the trace changes upon an increase in the concentration of the rotor, and the long-lived component increases in amplitude, we assign these changes to the dye aggregation.

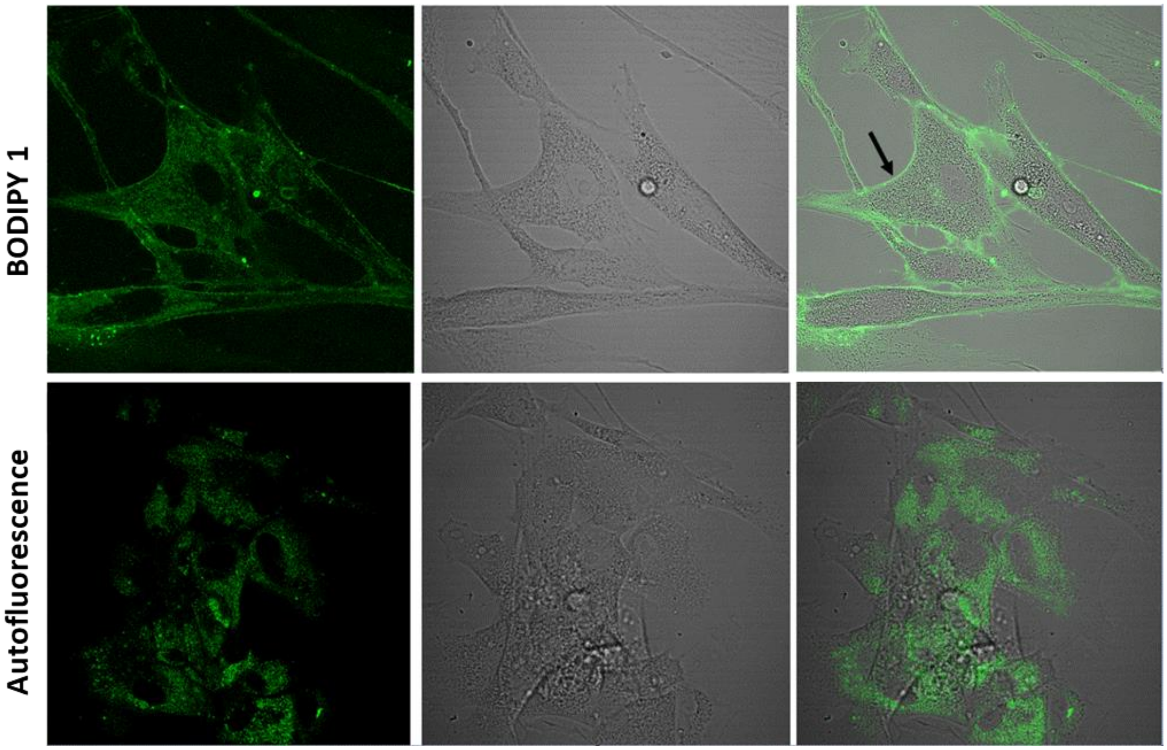

Figure S9. Fluorescence (left), brightfield (middle) and combined images (right) of MSCs: (top row) stained with 26.8 μM of BODIPY 1 for 30 min and (bottom row) incubated without BODIPY 1. The plasma membrane staining is clearly shown in the top series of images (e.g. a

location shown by the arrow). The autofluorescence signal is absent in MSCs plasma membrane regions, as shown in the bottom row (less than 0.01%). The image size is 213 × 213 μm.

2. BODIPY 1 staining

We used two channel detection FLIM: green channel predominantly recording the emission of the monomers and the red channel recording the emission of the aggregates (if present) to ascertain whether BODIPY 1 biexponential decay kinetics is caused by aggregation. It is well known that in the presence of aggregates, the red channel shows a very different decay kinetics, with a significantly larger contribution of the long decay component due to the aggregated species (4). In this present case, both channels show a very similar decay kinetics, which allows us to exclude aggregation of BODIPY 1.

a Membrane

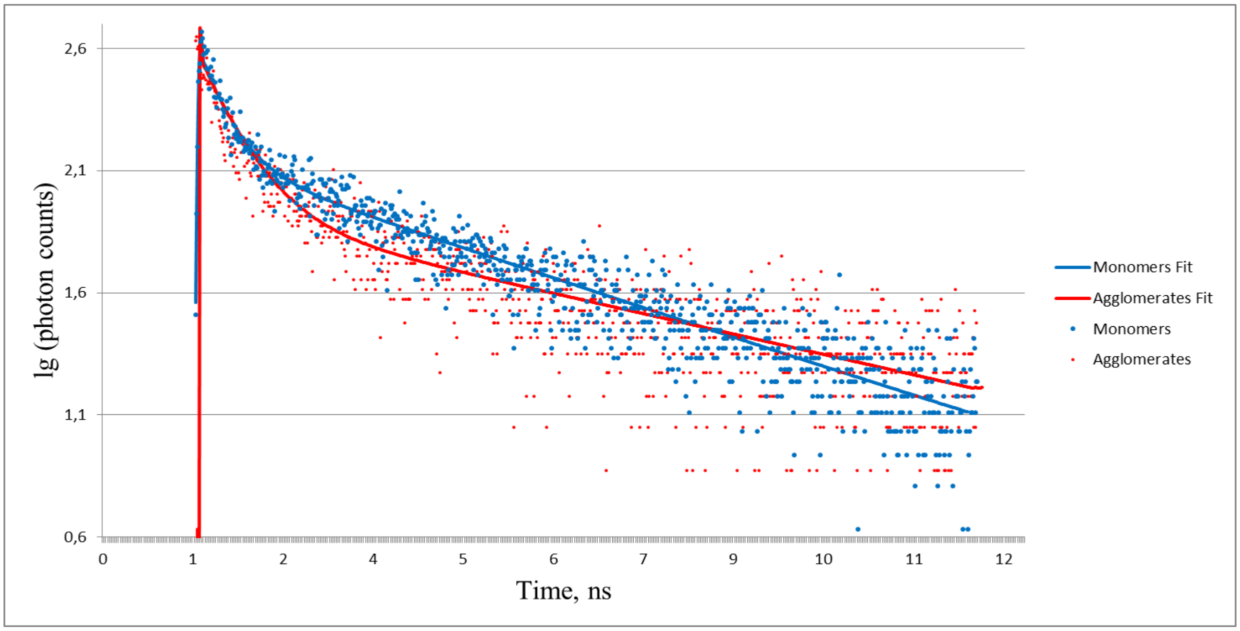

b Cytoplasm

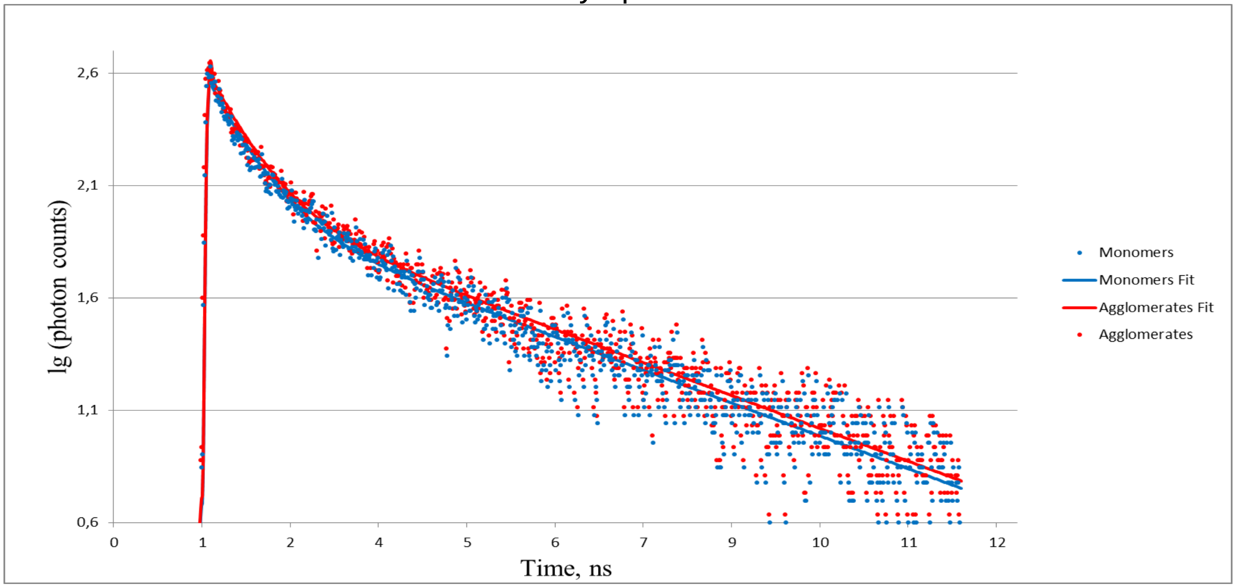

Figure S10. Time resolved decay traces recorded (a) in the plasma membrane and (b) in the cytoplasm of MSC cells stained with BODIPY 1 in the monomer channel (500–550 nm, blue) and the aggregate channel (598-660 nm, red). While small differences are detected in the case of the membrane traces, the fitted values are very similar. This data confirms that no significant aggregation of BODIPY 1 is taking place in these samples.

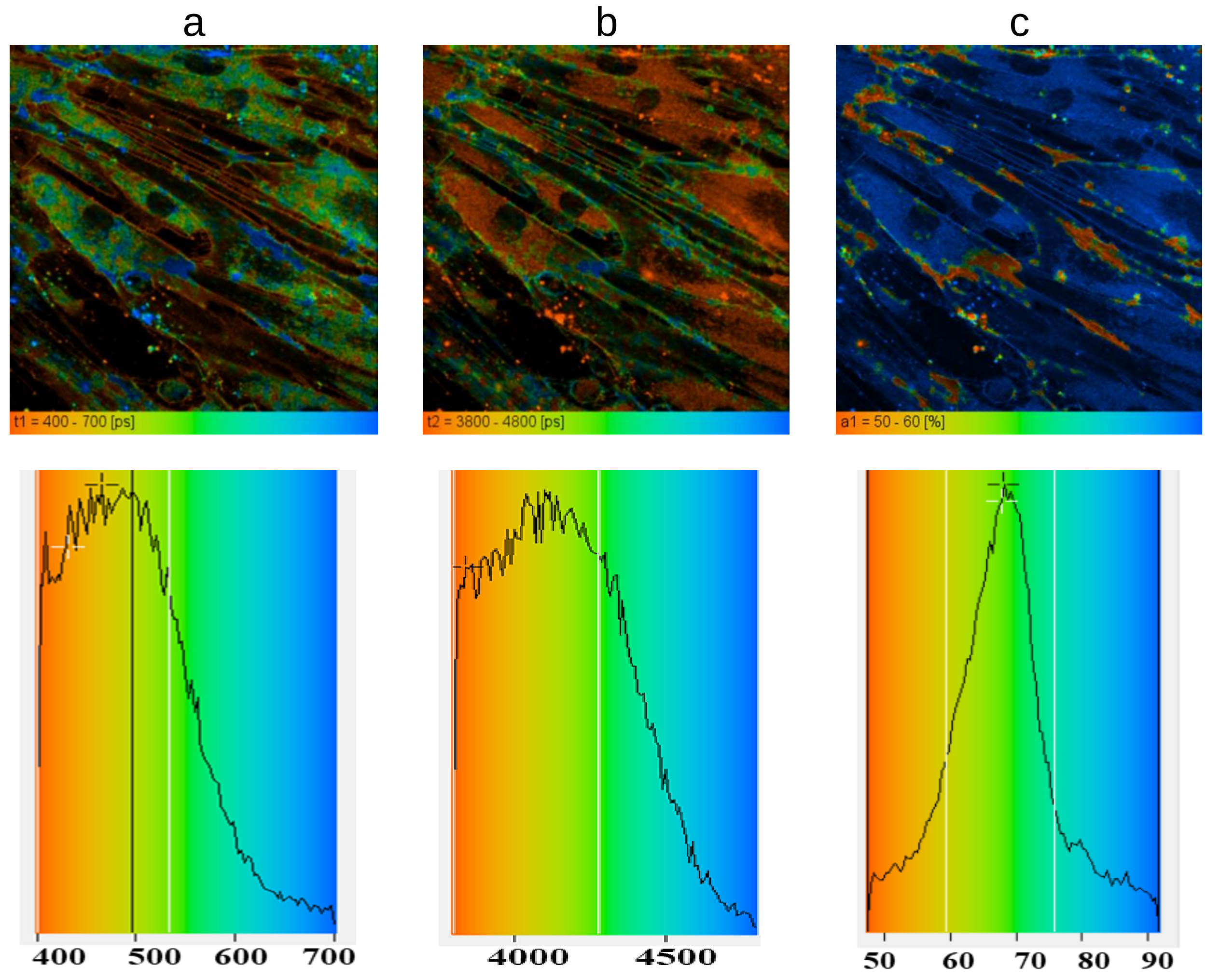

Figure S11. FLIM of control MSCs, stained with BODIPY 1. Pseudocolor-coded images and the corresponding histograms of the short τ1 (a) and long τ2 (b) components, and of the amplitude τ1 (c). The typical images are shown (top) along with the corresponding histograms (bottom). Field of view 213*213 μm (512*512 pixels). (SPCImage 5.7, https://www.becker-hickl.com)

Table S1. The parameters of the biexponential fit of FLIM data obtained with BODIPY 1 in MSCs during differentiation.

	**1 week differentiation**			**2week differentiation**			**3 week differentiation**		
	control	osteo	chondro	control	osteo	chondro	control	osteo	chondro
τ1,ns	389,84	598,38	487,45	516,84	558,55	464,28	475,07	500,75	668,5
	399,6	368,14	433,2	595,95	651,76	594,14	568,05	622,05	724,5
	514,45	386,49	365,82	499,5	530,19	372,83	511,3	571,95	655,54
	433,79	636,3	490,39	615,96	752,69	405,48	475,13	598,65	710,39
	433,05	600,98	437,87	491,23	665,64	407,97	436,4	589,72	689,31
	378,32	465,44	463,53	623,01	649,91	408,34	443,31	525,8	514,8
	337,68	556,98	470,23	519,97	613,48	734,22	439,19	609,77	689,31
	342,58	616,43	468,83	539,3	577,34	421,34	526,62	599,13	742,4
	330,91	661,61	438,01	611,36	574,82	477,59	447,6	539,24	686,75
	568,22	663,74	500,65	436,23	658,89	422,49	566,46	487,5	759,61
	449,33	473,46	609,8	483,76	561,57	464,56	590,97	682,9	784
	341,11	662,71	446,18	527,26	521,57	505,54	745,95	675,45	621,75
	421,18	452,27	519,32	544,33	665,1	458,5	620,71	631,73	535,18
	481,71	452,89	466,67	542,21	730,98	422,43	641,19	719,05	700,83
	399,17	397,23	559,62	511,45	658,03	455,94	680,55	585,4	759,61
	496,05	407,92	543,22	531,47	637,82	378,76	442,08	570,14	779,64
	336,4	671,02	378,39	561,61	710,27	414,76	541,5	647,31	798,3
	434,31	607,36	446,15	570,34	628,48	550,75	415	519,47	735,6
	368,96	503,31	431,14	606,26	509,22	468,74	371,65	543,83	721,68
	400,01	600,63	667,84	557	675,06	450,07	602,75	475,72	709,24
	552,77	371,66	362,72	487,91	502,27	433,49	532,42	591,19	496,46
	576,35	576,45	480,21	545,73	517,32	560,04	718,68	525,8	590,32
	534,28	345,55	498,48	573,15	543,5	449,12	551,07	622,8	515,83
	506,61	407,92	417,56	599,82	461,83	459,97	652,89	574,94	721,29
	474,89	402,17	369,27	521,39	530,05	497,27	683,42	603,64	605,65
	501,95	335,11	386,28	573	596,41	466,1	380,69	620,69	550,45
	513,76	347,84	460,93	539,24	565,16	602,79	475,33	546,38	656,98
	425,26	356,3	436,15	366,78	635,3	526,67	412,58	584,38	553,9
	580,25	577,33	494,23	375,86	556,65	525,87	459,82	738,7	737,35
	462,81	545,4	423,26	566,78	474,03	507	478,34	605,58	605,65
	552,77	622,41	384,45	491,23	521,97	603,22	480,01	694,43	670,61
	435,14	488,7	403,21	519,97	640,01	516,17	440,27	581,26	584,83
	484,75	473,22	495,09	617,07	546,99	641,82	519,69	488,83	540,01
	457,28	663,17	544,81	640,45	708,58	487,97	771,86	594,36	515,75
	500,13	655,14	457,45	580,46	664,99	497,81	558,66	677,33	550,45
	422,7	589,76	524,31	401,93	412,64	520,59	411,79	627,7	575,95
	484,12	569,69	512,99	635,24	410,21	522,76	381,98	600,68	625,02
	421,55	569,1	462,8	586,27	530,32	508,54	508,49	552,75	659,35
	465,66	520,96	467,19	631,29	495,95	563,96	644,85	708,55	667,49
	400,13	603,52	461,82	507,17	365,43	659,8	756,81	521,66	644,71
	410,89	593,55	357,88	467,77	440,95	608,19	643,83	585,83	616,03
	387,29	519,07	360,15	464,41	417,78	643,03	748,21	856,32	658,85
	425,47	649,38	536,89	560,03	599,8	402,83	678,01	615,69	720,55
	387,91	435,49	688,58	583,82	557,46	690,88	730,74	632,86	637,18
	402,96	449,12	487,89	586,55	766,5	508,28	552,15	527,93	560,5
	417,18	520,57	463,32	396,37	736,81	638,29	518,45	530,43	578,95
	431,02	525,36	366,62	449,7	484,16	611,24	540,27	645,6	598,74
	440,52	407,63	426,97	393,57	421,22	621,79	489,81	589,09	591,86
	486,21	720,47	506,15	442,66	530,3	534,27	633,77	642,94	542,69

	462,18	366,93	501,41	383,32	749,92	471,74	390,84	570,44	628,85
	471,77	353	394,74	335,71	420,05	619,06	510,02	630,42	600,59
	507,13	473,31	453,14	364,33	495,43	636,75	609,28	518,13	604,72
	443,65	348,22	475,62	457,11	661,88	630,29	437,85	583,54	710,04
	529,3	469,34	427,11	389,02	544,98	555,32	458,71	578,53	519,1
	447,09	346,68	460,26	392,36	616,03	537,52	466,35	492,57	490,55
	449,13	543,62	323,66	364,91	592,08	619,97	513,87	535,27	671,2
	478,76	392,19	442,99	624,46	468,9	460,27	634,86	775,89	694,42
	426,51	573,07	406,34	530,47	529,6	522,9	466,35	603,84	696,18
	374,68		490,73	361,17	549,82	509,25	678,87	575,38	719,74
	433,9		361,88	659,69	544,39	533,24	513,06	471,45	527,28
	386,76		439,6	504,06	600,95	544,47	420,95	592,74	798,59
	420,93		421,81	502,72	632,77	530,95	439	662,76	570,84
	384,52		437,16	488,19	386,18	475,27	685,18	522,5	503,33
	410,44		469,8	457,24	366,5	568,35	571,68	566,87	661,99
	392,44		550,28	404,34	556,75	539,72	572,26	677,82	545,59
	370,45		544,65	418,61	593,84	481,54	680,37	536	590,28
	469,68		439,22	351,53	504,15	473,15	556,82	792,06	699,46
	457,11		498,3	346,54	467,4	473,31	502,57	798,59	576,15
	501,81		528,81	601,77	465,64	451,09	478,78	785,52	537,69
	450,54		481,36	431,69	472,48	455,05	438,72	555,77	606,5
	349,36		485,12	406,79	488,7	606,98	463,2	446,35	684,33
	484,82		379,36	433,97	531,5	459,1	416,08	710,49	501,59
	437,87		483,36	374,9	479,12	502,51	442,67	620,79	531,69
	338,35		448,09	440,93	404,98	502,59	497,98	554,04	544,55
	430,38		531,92	412,34	479,27	423,07	457,07	762,09	522,67
	335,13		388,33	373,45	492,36	372,17	456,48	761,07	584,55
	384,63		453,28	453,09	668,42	520,02	475,54	684,8	486,12
	376,82		418,28	384,76	605,64	475,03	961,95	479,4	581,4
	309,93		504,31	363,14	673,64	415,16	672,42	616,02	585,17
	333,53		531,92	389,13	735,98	408,62	477,56	500,88	642,8
	349,35		416,37	375	628,53	462,31	745,6	534,53	484,78
	393,44		368,78	364,01	628,53	507,45	690,61	651,2	643,55
	392,92		362,46	321,1	650,38	542,82	681,8	505,51	547,22
	364,12		401,28	494,11	543,63	515,61	544,36	569,23	601,27
	440,86		462,5	508,88	560,72	471,69	716,79	595,51	564,05
	468,8		532,78	374,71	537,69	613,23	494,2	569,94	572
	368,12		516,71	510,17	676,33	384,47	412,76	775,54	750,87
	409,45		449,5	489,17	554,41	480,3	423,23	664,54	560,26
	387,19		448,5	485,42	547,74	499,48	392,37	557,81	765,15
	489,77		385,5	365,5	596,36	464,31	442,08	620,51	541,35
	374,72		481,62	610,49	712,67	464,82	443,49	683,48	514,62
			441,93	386,96		581,55		686,17	584,17
			450,71	266,69	632,49	422,45		758,82	591,6
			371,45	326,51	737,77	372,37		718,61	516,72
			491,95	585,77	591,73	532,73		559,46	511,22
			421,49	480,84	593,26	582,13		559,58	549,32
			446,39	411,84	697,87	631,54		509,7	522,27
			437,52	522,41	628,53	468,06		819,09	546,66
			411,28	362,17	551,41	483,62		721,05	563,88
			387,76	496,5	500,82	562,8		832,59	696,62
			387,32	584,74	639,04	463,08		734,01	548,76
			350,43	548,43	521,35	498,46		737,94	489,25
			431,23	374,08	489,73	549,67		803,72	591,96

			449,05	466,86	514,13	533,74		793,76	506,97
			368,71	388,32	448,84	403,74		759,9	572,18
			513,15	572,3	529,52	512,64		849,63	724,15
			385,82	556,77	516,3	528,11		836,13	556,53
			463,98	502,1	478,65	624,78		836,51	497,76
			413,68	478,4	513,96	587,16		702,04	563,88
			462,63	572,92	464,37	527,4		730,69	471,58
			401,41	435,18	577,42	610,78		711,19	454,26
			447,69	471,6	595,17	468,89		779,28	507,26
			448,66	398,16	523,54	657,88		784,57	651,56
			437,38	577,91	487,16	473,55		485,54	497,24
			374,06	552,23	413,77	454,12		680,87	594,45
			464,74	371,52	442,07	607,98		572,64	595,77
			436,73	402,13	606,51	637,07		581,34	631,79
			498,65	420,47	597,16	571,24		479,09	572,52
			417,47	418,84	551,97	653,53		709,89	497,9
			457,48	348,12	610,37	484,41		504,7	524,75
			539,44	408,74	540,5	555,59		640,87	691,97
			465,49	419,79	579,27	393,51		617,41	693,22
			477,7	412,55	452,02	470,52		573,43	510,94
			452,87	383,23	480,08	396,49		547,77	508,32
			417,46	480,36	384,21	488,51		764,16	571,2
			553,11	445,44	410,96	614,27		766,63	552,16
			546,62	456,08	450,36	494,92		634,64	542,7
			558,12	413,16	566,19	415,11		526,1	532,01
			530,82	517,14	575,51	580,08		510,82	573,89
			556,86	457,21	506,71	457,47		603,48	591,59
			556,02	330,84	481,75	394,88		653,42	536,41
			512,63	437,55	524,88	509,22		670,56	634,99
			549,23	446,83	452,4	558,33		782,47	600,99
			513,69	424,62	496,95	495,99		773,92	687,62
			556,71	556,29	504,25	621,49		506,08	559,31
			561,32	406,18	530,14	550,52		723,72	623,02
			560,35	454,29	471,26	591,36		523,06	618,19
			480,67	370,86	517,28	541,64		509,25	701,76
			511,3	446,3	391,59	560,91		807,08	795,05
			593,07	579,09	403,7	595,69		781,52	669,67
			575,36	492,05	502,34	582,7		609,07	599,56
			473,97	497,47	576,17	534,79		745,36	648,35
			542,88	484,5	624,59	647,85		677,8	485,08
			490,05	492,44	577,9	592,78		651,91	581,89
			438,43	454,29	533,59	484,99		801,01	668,74
			491,27	370,86	679,63	344,67		746,1	611,99
			533,97	448,92	524,95	587,73		680,72	616,18
			534,94	445,01	426,46	544,79		804,35	701,84
			425,12	365,39	590,06	523,26		769,52	505,19
			390,29	369,51	469,8	489,61		725,15	622,79
			330,84	445,44	663,56	647,28		621,53	589,94
			523,85	442,07	568,45	577,15		737,33	578,31
			512,46	419,46	490,2	540,28			603,88
			372,48	485,36	445,92	558,63			662,51
			355,86	497,34	381,37	616,52			576,71
			433,11		391,07	493,49			574,62
			396,97		376,51	432,08			507,85

			385,46		420,11	489,07			492,29
			436,23		394,26	518,86			514,66
			428,66		497,51	540,15			634,23
			499,65		524,95	490,21			544,86
			516,97		536,65	515,55			687
			557,23		390,99	641,02			636,9
			469,31		465,87	542,52			679,66
			475,3		530,47	499,9			596,02
			350,58		386,78	653,12			567,61
			386,37		491,06	609,08			674,45
					583,22	648,4			656,12
					530,45	653,27			493,52
						560,41			568,69
						512,24			537,65
						632,91			668,85
						691,92			642,42
						624,45			614,09
						609,6			750,27
						552,63			582,09
						589,22			672,41
						570,66			673,66
						644,19			703,98
						653,83			652,16
						527,59			710,46
						609,07			570,04
						625,75			720,41
						543,16			649,03
						546,88			641,81
						591,23			472,69
						630,8			495,5
						627,47			573,29
						605,81			609,48
						450,41			568,5
						454,29			693,59
						534,12			552,6
						487,91			567,73
						471,4			598,04
						537,32			661,76
									646,4
									585,1
									607,72
									582,05
									515,95
									588,57
									598,67
									555,59
									521,95
									646,28
									572,95
									639,13
									706,7
									487,51
									662,05
									480,29

									613,88<br>616,91<br>548,44<br>489,31
**Mean τ1**	**431,732 ± 61,53**	**508,477± 109,10**	**461,99± 65,02**	**471,796 ±84,5**	**543,644 ±92,2**	**527,83 ±77,71**	**541 ± 115,82**	**636,8 ±101,3**	**605,5±77,8**
τ2,ns	4340,6<br>4113,7<br>4182,2<br>4039,4<br>4089,7<br>4071,4<br>3944,5<br>3972,2<br>3832,4<br>3897,5<br>3809<br>3887,5<br>4381,6<br>4277,1<br>4231,8<br>4240,1<br>3952,3<br>3941,9<br>4219<br>4298,5<br>4157,6<br>4240,1<br>4241,1<br>4471,9<br>3960,9<br>4290,4<br>4406,7<br>4056,2<br>4436,2<br>4264<br>4157,6<br>3608,4<br>3629,4<br>3989,9<br>3447,7<br>3411,8<br>3828,3<br>3371,1<br>3685,7<br>3473<br>3508,7<br>3524,8<br>4193,5<br>4093,8	4591,9<br>3643,9<br>3814,5<br>4214,2<br>4557,8<br>4356,4<br>4209,3<br>4650,5<br>4500,9<br>4316,2<br>4034,6<br>4661,8<br>4952,7<br>4308,9<br>4183<br>3994,2<br>4045,5<br>4055,5<br>4217,9<br>3832,6<br>4395,8<br>3988,5<br>4249,8<br>4471<br>4668,6<br>4338,6<br>4110,4<br>3956,4<br>4232,4<br>4130,5<br>4609,8<br>4086,5<br>4168,7<br>4487,2<br>4125,4<br>4462,5<br>3978,7<br>4018,4<br>4275,5<br>4260,6<br>4337,7<br>4656,7<br>4194<br>4184,7	3990,1<br>3900,8<br>3886<br>4005,3<br>4325,7<br>3875,6<br>3999,1<br>3875,9<br>4087,7<br>3945,8<br>4117,7<br>3936,9<br>3885,3<br>3828,7<br>4339,7<br>4305,6<br>3660,8<br>4016<br>4216,6<br>4295,6<br>4069,3<br>3785,7<br>3998,8<br>3702,2<br>4287,9<br>3870<br>4002,1<br>4235,2<br>4254,2<br>3739,8<br>3913,9<br>3650,2<br>3921,2<br>4168,9<br>3382<br>4154,1<br>3935,6<br>4113,7<br>3815,6<br>4098,3<br>3683,6<br>4086,8<br>4465,1<br>4517,8	4108,2<br>4159,3<br>4285,6<br>4058,3<br>3430<br>4481,9<br>3746<br>4051<br>3950,5<br>3810,9<br>3443,1<br>4154,8<br>4019,6<br>4475,6<br>4253,1<br>3557,1<br>4346,6<br>3726,1<br>4375,3<br>4330,4<br>3952,5<br>4152,8<br>4104,1<br>4104,7<br>4071,3<br>3953,8<br>4002,1<br>3457,3<br>4404,3<br>4665,6<br>3430<br>3746<br>4179,8<br>4250<br>4279<br>4304,2<br>4503,5<br>4177,6<br>4663,5<br>3537,4<br>4234,2<br>4321,4<br>4058,9<br>4156,9	4212,7<br>3989,1<br>4183,2<br>4468,4<br>3949,5<br>4442,9<br>4174,6<br>3832,4<br>3529<br>4471,4<br>3995,7<br>3547,5<br>4057,6<br>3999,7<br>3190,9<br>3786,9<br>3588,4<br>4204,8<br>4057,6<br>4298,1<br>3803,8<br>4044,7<br>4036,1<br>3928,8<br>3736,1<br>4103,6<br>3841,9<br>3679,7<br>4061,2<br>3536,6<br>3967,7<br>4040,2<br>3956,2<br>4423,7<br>4400,7<br>4554,2<br>4426,3<br>4271,4<br>4434,9<br>3510,3<br>4593,7<br>4525,8<br>4405,8<br>4208,3	3472,6<br>4187,3<br>3268,5<br>3860,8<br>3503,6<br>4213<br>4499<br>3765,6<br>4158,9<br>3979,1<br>4028,2<br>4220,9<br>3924,1<br>4158,5<br>3943,2<br>3775,3<br>4061,3<br>4132,9<br>3970,8<br>3980,9<br>3535,4<br>3631,5<br>3850,1<br>4206<br>4086,5<br>3916<br>4406,4<br>4273,2<br>4060<br>4169<br>3636,1<br>3751,4<br>4290,1<br>4296,3<br>3848<br>3938,7<br>3892,7<br>3977,1<br>4135,2<br>4180,5<br>4348,5<br>3949,6<br>3252,5<br>4313	3735,4<br>4185,6<br>4234,6<br>4139,8<br>4644,8<br>4106,6<br>5471,6<br>4943,5<br>5355,7<br>5188,7<br>5414,8<br>5154,2<br>5411,6<br>4513,1<br>5467,2<br>4942,1<br>5529,8<br>3990,1<br>5774,7<br>5177<br>4400,8<br>5056<br>4337,9<br>5236,6<br>5138,9<br>4632,5<br>4328,9<br>4948,9<br>5325,8<br>5014,7<br>5121,1<br>5545<br>5416,7<br>3893<br>4440,3<br>4646,9<br>4860,9<br>5174,8<br>5494,5<br>5002,1<br>4614,6<br>5151,4<br>4978,6<br>4668	4537,4<br>4603,2<br>4940,5<br>4917,8<br>4875,2<br>4753,7<br>4700,6<br>4632,4<br>4480,1<br>4672,8<br>4607,2<br>4407,8<br>4875,8<br>4755,9<br>4257,3<br>3911,7<br>4280,4<br>4161,4<br>4133,1<br>4890,5<br>4875,2<br>4923,4<br>4659,4<br>4906,7<br>4681,5<br>4894,1<br>4887,4<br>5234<br>4996,2<br>4705,2<br>4505<br>4739,5<br>4698,7<br>4744,6<br>4084,3<br>4125,3<br>4178,8<br>4805,8<br>3956,5<br>4510,9<br>4877,9<br>4533,8<br>4425,1<br>4029,6	4053,8<br>5183,9<br>4751,1<br>4903,1<br>5351,8<br>4288,9<br>5351,8<br>5102,6<br>4294,5<br>4835,5<br>4420,6<br>4382,6<br>3944,4<br>4892,5<br>4835,5<br>4996,6<br>4118,5<br>4774<br>4964,5<br>4446,5<br>4085<br>4572<br>4050,8<br>4562,8<br>4722,3<br>4277,5<br>4799,3<br>4492,4<br>5067,3<br>4722,3<br>4813,9<br>5193,4<br>4583,9<br>4653,9<br>4277,5<br>4462,8<br>4584,2<br>4830,7<br>4590<br>4535,5<br>4353,7<br>4648,6<br>4620,6<br>4945,2

	4209,2	4547	4458,2	4174,9	3668	4268,5	4993,3	4270,8	4132,9
	4177,9	4037,8	4236,6	4219,3	4247,6	4252,5	4408,6	4674,7	4754,6
	3776,5	4031,7	3990,4	4181	4559,8	4272,9	4394,9	4397,4	4886
	3980	4161,6	3907,3	3968,2	4683,3	3636,9	4355,5	4727,4	4847,5
	4249,5	3826,8	4166,1	4178,4	4360,3	3548,5	3900,2	4205,7	4661,9
	4267	4310,9	4055,3	4363	4709,5	3910,1	3692,5	4895,6	4671,8
	4406,4	4090,8	3537,9	3998,2	3583,4	4306,2	4774,8	4255,9	4955,7
	4100,5	4666	3464,5	3807,9	3574,1	4202,9	4852,3	4549,8	4718,9
	4079,6	4180,4	4330,1	4576,9	4044,9	3902	4505,6	3927	5113,3
	4243,2	4285,5	4218,1	4437,6	3432,1	3592	4369,1	4794,5	5058,9
	4031,9	3948,4	4268,3	4150,3	3872	3433,4	4164,5	4969,4	4324,5
	4103	4331,1	3355,5	4223,3	4097,8	4091	4825,6	4857,1	4704,7
	4093,8	4417,3	3981,1	4287,8	4150,8	3446	5203	4283,7	5231,7
	4326,3	4318,4	3590,6	4854,6	3963,5	3811,9	4164,5	3954,1	4814
	4368,3		3585,4	4498	4048,1	3301,1	4641,6	4351,2	5219,1
	3793,4		3398,8	4590,4	4423,9	3625,3	3926,6	3843,4	4501
	3555,7		3957,3	4387,4	4695	3426,2	4936	3971,1	5054,9
	3911,8		3590,4	4247,5	4137,3	4012,1	4519,4	3857,3	4768,2
	3615,7		3893,4	4600,1	3548,1	3550,4	5655	4555,1	4593,3
	3915,7		4220,9	4766,6	3510,5	4199,8	4743,5	4694,2	4961,2
	3631,1		4146,9	4357,1	3602,1	4089,2	5075,2	4526,8	4394,9
	3338,8		4162,6	4448,3	4032	4030	4570,6	4861,9	4240,4
	4092,9		3979,4	4561,5	3717,3	4322,3	4376,6	5021,9	4488,2
	3020,6		3961,1	4153,7	3851,5	3999,4	3964,8	4341,6	4420,5
	3822,5		3194,3	3620,4	4071,8	3717,8	4679,3	4525,3	4139,5
	3974,2		3679,2	4041,4	3291,3	4282,9	4846,3	4027	4084,7
	3970,2		3617,2	4155,7	3842,8	4647,1	5131,9	4766,6	4569,9
	4278,1		3189,5	4147,6	3968,6	3924,1	4688,7	4186	4190,2
	4106		3540	4021,6	3605,7	4207,4	5038,4	4720,4	4614,5
	4283,5		4188,7	4121,6	3729	4072,6	4248,8	4666,7	4079,4
	4081,9		4247	4083,9	3958,4	3829,7	4690,8	4149	4216,3
	4208,5		4154,3	4106,6	3796,2	4120,9	5092,5	4337,2	5192,5
	3920,5		4047,9	4065,4	4069,8	4177,2	4884,9	4058,8	4825,6
	3772,3		3982,2	4258,2	4254,5	4322,3	4432,6	4677,5	4730,7
	3987,1		4102,1	3735,3	4342,2	3581,6	4475,1	4222,6	4744,1
	4102,8		4247	3540,4	4261,4	3142,7	4235,2	4140,9	4583,6
	3884,1		3778,3	3907,5	4204,7	4028,6	4985,7	4743,7	4282,7
	4190,6		3637,5	3705,8	4204,7	4184,6	4749,7	4482	4848,1
	3841,1		3569,5	4311	3888,7	4285,2	4405,9	4676,7	4335,1
	4007,3		4190,5	4539,9	3581,8	4056,4	3934,2	4438,4	4800
	4261,4		4119,1	4798,2	4105,8	4012,2	4607,7	4468	4507,7
	4087,7		4059,5	3410,5	3913,6	4281,4	4717	4925,6	4871,9
	3743,3		4039,3	3893,6	3546,3	4055,2	4824,4	4395,1	4641,2
	4085,2		4049,4	4118,4	3526,4	3671,5	4396,5	4258,4	4176,7
	3842,9		4194,8	4164,3	3956,9	4077,2	4788,3	4183	5012,1
	3807,8		3951,5	3917,6	4052,9	3614,4	5141,5	4819,5	4419,8
	4101,8		4125,2	4499,9	4205,5	3793,7	5817	4937	4352,9
			4087,8	4103	4308,6	4038,6		4764,6	4204,9
			3846,3	4099,7	4198,3	3386,1		4712	4464,1
			3184,1	4103,4	4252,2	3364,1		4151,3	3864,4
			4210,8	4376,9	3855,3	3867		4136	4003,5
			3880,8	3650,2	3722	3549,4		4052,2	4289,7
			3970,4	3664,3	4321,8	4011,1		4864,5	4533,4
			3651,4	4539,1	4204,7	3627,1		3907	4974,6

			3687,7	3843,4	4012,3	3920,6		4566	4507,2
			3932,7	4604	3844	3769,1		4211,4	5007,7
			3691,8	4302,9	4015,4	3773,3		4868,5	4286,8
			3955,7	4237,3	3931,8	3602,2		5273,8	3967,5
			3812	3473,5	3885	4008,2		4985,2	4869,7
			4060,9	4294,8	4205	3928,6		4412,6	4222,8
			3045	4191,7	3839,5	3717,8		4840,9	4593
			4262,1	3587	4132	3488,6		4664,3	4829,2
			3822,3	4291,7	3923,4	3657,6		4356,3	5037,5
			4000,8	4329,6	3831,7	4323,3		4223	3955,1
			3953,2	3891,3	3936	4288,2		4840,4	4507,2
			4121,6	4624,1	3903	4207,4		4246,1	4635,1
			3517,9	3740,1	3649,2	4303,8		4227	4718,1
			3822,9	3831,6	3868,7	3761,8		4867,6	5013,4
			3862,1	3573	4056,2	4295,1		3903,5	5011,5
			3959,2	4529,8	3985,8	3706,4		5128,1	4620,3
			3971,4	3981,3	3588	3298,9		4254	4260,8
			4227,9	4034,5	3705,1	4204,9		4923,8	4881,2
			3652,7	4704,1	3843,7	4186,5		4287,6	4183,1
			4038,5	4697,1	4048,8	3666,3		5125,8	4690
			4099,3	3683,3	3886,7	4217,3		4069,9	4702,2
			4196,6	4383	4082,7	3832,4		4514,3	5046,3
			4701,4	4315	4186,6	4237,6		4415,6	4796,5
			3585,9	3865,1	4129,1	3936,8		4334,9	5029,3
			3891	4201,5	3406,2	4019,1		4603,8	4298,3
			4042,3	4375,4	3721,5	3747,2		4889,7	4339,7
			3525,5	4445,2	3333,4	3920,1		4979,4	4531
			4293,1	4160,2	3831,8	3914,1		4339,8	4784,6
			4125,9	4305,9	3865,9	4078,7		3862,1	4774
			3538,9	4549,3	3787,2	4050,8		4650	4537,5
			3901,8	4504,9	3887,5	4461,4		4533,4	4640,3
			4191,5	4289,6	4160	3978,6		4673,2	5078,5
			4005,4	4220,3	4038,6	3243,7		4936,7	5009,2
			3982,5	3959,5	4003,5	4128,4		5021	4529,5
			3783	3514,6	3949,3	4236,9		4540,9	4906
			4201,9	3724,5	4053,6	4141,5		4593,2	5085,9
			4034,3	4019,9	3923,6	4031,3		5016,3	5013,8
			4228,6	3611,7	4193,7	4352,4		4967,6	4404,9
			4375,5	4272,4	3571,2	4206		5246,4	4571
			3628,2	4093,5	4285,8	3907,7		4731,3	4970,9
			4276,7	3946,3	3395,6	4054,7		5028,1	5222,1
			4278,2	3479,2	3104,8	4207		4636,9	4772
			4076,3	4315,5	3933,5	4132		5100,9	4123,1
			3514,6	4078,9	3865,8	3717,8		4733,2	4367,5
			3702,9	4264,5	4062,8	3909,6		4802,5	4501,3
			4098,8	3854,8	4247,6	4375,3		5172	4601,2
			3872,3	4272,4	4132,9	4119,2		4579,5	5130,7
			4160,7	4093,5	4845,7	3131,1		4951,2	4919
			4017,8	3693,3	4580,6	4179,9		4890,8	4753,7
			4171,2	3570,9	4188,8	4314,7		5096,3	4839,3
			4243,1	4331,5	4001	4042		4653,1	4606,8
			3943,8	4429,3	3636,5	4078,6		4765,7	4493,3
			4274,1	4160,2	4040,1	3940,6		4858,4	4862,3
			4309,5	4278,6	4371,8	3908,6			4742,2

			4230,5<br>4093,7<br>3777,2<br>4298,7<br>4236,1<br>4311,9<br>3874,8<br>3972,9<br>4164<br>4259,6<br>4239,5<br>4222,9<br>3975,2<br>3358,1<br>3448,6	3860,5<br>4518,2<br>4316,6	3725,1<br>4150,4<br>4113,2<br>3970,7<br>3552,5<br>4353,4<br>4223,6<br>4348,8<br>4580,6<br>4521,8<br>3441,1<br>4345,3<br>4360,2<br>3660,9<br>4396,7<br>4431,9<br>4108,1	3839,9<br>3705,6<br>4799,3<br>4536,8<br>3854,4<br>4251,9<br>5043,4<br>4143,1<br>4132,9<br>3647,7<br>4821,6<br>4370,2<br>3949,2<br>4047,9<br>4280,4<br>4160,4<br>4340,2<br>3310,7<br>3663,3<br>4338,3<br>4067,6<br>3342,2<br>3757,4<br>3442,3<br>3371,1<br>4106<br>4225,8<br>3916,8<br>3835,2<br>3806,1<br>3318<br>3983,6<br>3929<br>3681,9<br>3961,9<br>4030,7<br>4277,3<br>3454,9<br>3879,9<br>3869,7<br>3261,2<br>3671,2<br>3395,8			4687,5<br>4411,6<br>4248,8<br>4443<br>4061,8<br>4158,3<br>4257<br>3883,8<br>4397,4<br>4932,1<br>4637,4<br>4294,8<br>4452,5<br>4412,3<br>4956,7<br>4509,6<br>4325,2<br>4235,7<br>4616,1<br>4726,5<br>4841,6<br>4648,2<br>4779,5<br>4177,4<br>4211,5<br>4420,4<br>4394,9<br>4965,3<br>4852,4<br>4431,3<br>4742<br>4597,2<br>4446,9<br>4470,1<br>4200,4<br>4358,6<br>4581,7<br>4289,8<br>5150<br>4296,6<br>4257,3<br>4424,9<br>4601,3<br>4810,7<br>4522,7<br>4421,6<br>4614,9<br>4815,6<br>4853,9<br>4507,8<br>4615,3<br>3959,7<br>4173<br>4796,6

									4845,9<br>4840,1<br>4392,6<br>4327<br>4358,3<br>4580,2<br>4328,4<br>4456,8<br>4578,7
**Mean τ2**	**3998,03 ±283,38**	**4253,2 ±257,67**	**3968,85± 284,80**	**4128,58 ±326,2**	**4008,4 ±324,54**	**3953,30 ±331,24**	**4754,24 ±487,27**	**4575,8 ±345,48**	**4599,0± 317,8**
α1, %	76,2<br>72,06<br>75,81<br>76,59<br>74,88<br>79,13<br>77,12<br>77,41<br>72,92<br>62,92<br>59,27<br>62,58<br>62,17<br>61,75<br>73,52<br>67,88<br>75,5<br>75,95<br>72,78<br>66,55<br>65,77<br>69,88<br>71,85<br>67,77<br>72,85<br>73,19<br>74,98<br>72,13<br>57,74<br>63,23<br>65,77<br>81,71<br>67,75	64,23<br>49,82<br>46,5<br>47,06<br>57,01<br>49,57<br>48,49<br>46,86<br>44,91<br>38,48<br>37,61<br>41,42<br>58,91<br>53,84<br>58,88<br>56,07<br>37,81<br>38,79<br>45,65<br>48,2<br>64,06<br>39,38<br>54,21<br>42,37<br>66,5<br>63,72<br>52,31<br>60,14<br>42,52<br>41,52<br>53,54<br>49,68<br>54,4	65,43<br>64,45<br>69,67<br>62,93<br>62,59<br>62,11<br>65,86<br>64,64<br>67,77<br>66,26<br>59,31<br>68,36<br>65,24<br>66,86<br>66,12<br>66,87<br>71,66<br>72,92<br>60,84<br>66,25<br>64,2<br>65,11<br>62,75<br>65,03<br>74,8<br>74,44<br>73,45<br>66,53<br>67,45<br>67,4<br>73,91<br>73,66<br>64,59	58,58<br>61,03<br>63,06<br>60,33<br>55,94<br>60,15<br>57,59<br>58,73<br>54,97<br>60,62<br>55,01<br>57,71<br>63,15<br>63,39<br>67,04<br>60,1<br>63,95<br>51,21<br>59,6<br>64,44<br>56,64<br>58,96<br>66,06<br>57,46<br>65,55<br>63,49<br>49,13<br>74,95<br>63,48<br>59,15<br>55,94<br>57,59<br>56,19	50,56<br>43,68<br>50,31<br>50,36<br>39,68<br>55,48<br>55,45<br>55,91<br>49,14<br>41,01<br>41,47<br>35,65<br>54,48<br>44,22<br>35,51<br>42,79<br>34,1<br>52,51<br>59,68<br>54,08<br>55<br>59,49<br>61,15<br>65,64<br>62,96<br>67,36<br>67,15<br>42,92<br>52,6<br>56,62<br>50,82<br>67,62<br>66,12	60,65<br>63,57<br>64,79<br>67,05<br>65,12<br>64,6<br>67,03<br>72,95<br>75,39<br>74,35<br>71,37<br>67,85<br>69,79<br>69,04<br>68,37<br>67,05<br>66,97<br>65,45<br>61,83<br>73,06<br>67,32<br>57,47<br>63,17<br>65,13<br>67,34<br>70,54<br>62,69<br>65,86<br>63,45<br>63,27<br>60,54<br>61,21<br>64,37	79,95<br>80,9<br>73,84<br>78,74<br>74,46<br>75,92<br>58,97<br>40,62<br>51,76<br>52,1<br>48,73<br>43,95<br>55,23<br>66,85<br>54,83<br>62,29<br>60,08<br>70,54<br>66,33<br>52,79<br>58,78<br>56,07<br>55,59<br>59,43<br>54,04<br>65,1<br>54,08<br>58,11<br>63,28<br>67,61<br>64,11<br>68,6<br>63,38	66,85<br>69,02<br>64,24<br>70,59<br>69,24<br>69,12<br>67,11<br>67,97<br>66,49<br>67,29<br>64,75<br>63,7<br>62,15<br>65,79<br>66,4<br>64,2<br>55,16<br>65,37<br>66,2<br>67,49<br>69,12<br>69,12<br>68,03<br>67,11<br>66,77<br>67,68<br>65,93<br>66,03<br>67,79<br>68,01<br>62,51<br>65,3<br>64,27	57,35<br>59,63<br>59,04<br>57,69<br>64,22<br>58,76<br>64,22<br>61,36<br>53,71<br>57,52<br>58,5<br>59,72<br>55,82<br>62,04<br>57,52<br>58,54<br>50,71<br>60,31<br>56,55<br>53,95<br>56,99<br>58,66<br>58,73<br>57,41<br>59,35<br>60,18<br>59,69<br>58,78<br>60,83<br>59,35<br>57,1<br>67,32<br>62,92

	72,89	51,12	69,53	62,23	43,51	65,02	38,71	66,3	60,65
	71,32	42,68	64,13	48,41	42,65	68,18	46,8	61,73	60,18
	75,07	49,93	63,43	68,25	56,45	70,05	52,71	57,69	61,89
	75,33	42,45	67,44	42,46	52,95	68,96	70,95	58,48	55,86
	73,72	43,29	70,06	35,31	58,31	61,87	50,31	60,33	61,51
	69,09	50,58	66,11	49,21	62,18	64,45	55,55	61,83	60,01
	78,05	45,18	68,24	53,18	48,47	59,75	41,82	60,86	59,7
	82,38	45,21	71,43	62,94	61,23	63,2	43,41	62,39	60,55
	79,09	49,41	74,08	57,47	61,34	59,71	48,97	65,85	58,07
	71,39	44,35	69,69	60,86	44,15	65,22	50,28	61,04	55,67
	70,11	51,24	63,15	62,37	43,23	64,98	44,64	63,62	62,8
	70,79	54,99	68,36	58,86	33,39	61,67	69,91	63,29	62,41
	73,44	51,36	64,83	60,53	40,75	67,04	66,89	63,81	65
	68,58	44,8	67,4	59,41	52,36	63,31	63,66	64,81	60,99
	73,88	60	65,58	60,2	55,54	57,39	64,73	61,21	60,81
	68,65	44,05	63,42	56,99	47,02	60,16	52,38	67,67	63,1
	72,38	57,61	67,87	61,32	43,09	61,28	74,04	62,73	61,08
	73,92	53,18	65,52	62,14	58,2	62,05	72,33	66,27	64,47
	66,33	52,41	58,24	47,78	41,92	64,85	67,5	55,89	62,79
	73,45	59,05	74,45	60,73	56,21	61,63	72,87	62,75	60,42
	75,2	57,24	73,16	64,03	47,29	61,33	69,05	60,89	68,99
	76,24	59,13	72,11	58,56	45,12	57,76	47,31	68,83	67,81
	80,57	49,91	69,82	61,17	53,55	59,77	47,09	67,93	66,72
	77,79	56,65	70,41	58,21	60,08	60,22	57,24	64,1	65,59
	74,97	41,4	70,53	58,63	53,29	58,94	47,31	62,79	61,73
	73,74		62,34	62,82	56,86	54,29	56,58	57,48	67,23
	79,44		61,52	55,21	60,97	58,57	49,28	59,72	63,73
	82,86		70,88	66,64	57,05	56,62	58	50,59	60,3
	82,62		64,46	66,39	46,2	60,18	48,64	55,31	62,48
	78,88		68,41	66,76	59,67	56,7	54,77	62,48	64,67
	74,92		59,56	68,29	60	64,98	39,53	61,92	59,01
	76,06		62,5	68,05	52,91	66,8	64,94	62,01	62,48
	73,6		62,57	68,32	58,31	61,24	57	63,73	61,36
	77,29		63,23	73,2	60	67,81	45,62	59,37	62,62
	70,07		62,62	70,45	65,66	63,49	42,78	61,35	61,69
	73,57		54,09	36,83	66,07	65,75	49,57	57,66	58,41
	77,71		58,96	58,29	60,91	69,41	53,77	56,36	54,01
	79,23		59,88	64,07	66,93	66,89	58,28	59,77	55,49
	69,78		60,87	63,1	67,45	64,97	59,34	59,63	56,93
	70,05		64,1	64,43	64,96	60,51	58,69	60,9	56,75
	75,47		65,45	60,49	64,15	65,45	45,33	59,94	57,93
	65,9		65,46	59,43	63,52	65,88	43,73	52,45	61,89
	69,3		60,18	56,26	63,54	71,61	57,48	57,01	62,82
	66,28		53,12	51,71	57,4	66,14	53,13	54,95	64,96
	73,14		61,13	63,08	58,74	68,17	45,09	58,91	61,03
	75,42		65,41	50,18	60,6	71,51	46,31	60,89	63,68
	75,45		65,46	60,67	54,91	64,85	54,04	59,22	63,29
	76,81		72,57	65,74	56,23	57,01	53,11	57,26	57,72
	75,09		73,7	67,33	56,23	60,13	50,5	60,13	58,28
	72,93		66,07	63,69	56,38	70,63	51,37	53,03	57,68
	73,69		69,43	65,84	55,02	64,18	45,07	59,35	62,21
	73,12		63,84	65,35	60,81	63,57	46,06	62,49	61,28
	74,29		61,81	45,33	56,28	60,35	74,68	64,57	65,46
	72,06		61,09	58,22	50,52	60,38	75,07	60,43	60,6

	65,23		61,13	44,06	55,62	57,65	73,09	70,5	54,73	
	72,64		65,15	55,1	61,09	60,08	79,01	62,8	60,88	
	58,83		64,76	49,2	58,4	63,11	70,94	69,22	61,35	
	75,44		64,05	60,37	60,64	60,23	64,77	63,76	60,3	
			60,55	55,59	63,56	65,54		61,65	57,51	
			60,36	62,25	64,25	64,14		58,83	57,68	
			64,53	66,97	60	63,8		56,6	55,25	
			67,03	59,31	63,35	60,03		57,09	60,92	
			65,45	38,66	50,46	46,27		59,42	60,72	
			62,2	37,7	58,97	49,67		61,34	66,81	
			61,37	50,16	56,23	55,65		61,44	66,67	
			58,47	52,94	60,1	60,84		55,9	63,28	
			51,32	59,99	57,89	60,81		57,82	59,23	
			70,72	48,35	56,48	64,56		59,38	63,4	
			75,8	45,78	61	62,71		65,52	59,6	
			70,77	56,78	56,27	65,39		62,52	64,15	
			73,27	58,27	63,41	63,5		62,03	61,37	
			67,06	49,57	62,36	65,98		64,15	58,65	
			70,52	41,69	63,14	57,76		55,91	60,46	
			71,8	54,18	62,19	58,62		53,09	67,59	
			65,03	50,65	61,08	66,62		55,81	59,58	
			67,77	40,85	63,84	64,1		60,54	63,28	
			69,53	48,74	64,84	67,81		60,83	72,84	
			60,59	40,22	62,69	67,18		60,23	68,37	
			62,97	43,92	64,4	64,9		62,75	68,15	
			63,54	43,46	64,57	62,52		62,74	57,22	
			67,79	47,98	66,61	66,52		55,44	59,18	
			70,06	44,32	64,97	55,79		58,8	61,37	
			70,36	55	63,22	56,95		58,27	63,24	
			61,78	56,54	64	59,09		62,69	55,27	
			68,09	62,74	64,4	55,39		57,21	62,08	
			65,5	43,22	61,65	64,1		49,72	59,21	
			62,31	61,68	63,59	62,8		59,96	65,71	
			65,2	54,95	66,25	66,79		61,81	53,54	
			60,05	42,36	63,26	69,62		57,89	64,77	
			62,44	45,67	57,98	68,73		60,09	61,7	
			65,45	48,25	62,44	68,74		60,66	59,84	
			61,23	47,64	55,78	64,98		46,69	60,65	
			65,13	42,41	60,65	61,72		48,74	60,35	
			63,23	48,21	56,72	67,31		40,97	64,08	
			57,33	51,54	56,59	68,61		53,13	63,5	
			52,99	55,76	57,19	69,63		57,26	65,63	
			66,57	59,58	63,26	65,06		54,53	67,67	
			65,2	63,4	66,81	63,81		35,02	68,54	
			63,21	64,11	59,7	68,74		41,36	61,09	
			64,24	60,98	61,69	63,95		37,67	56,95	
			68,09	63,02	60,28	65,22		37,68	61,37	
			67,57	60,05	61,87	65,41		46,51	60,85	
			56,98	54,68	66,38	66,09		50,55	59,51	
			61,54	58,73	61,45	65		66,23	53,41	
			54,53	57,78	64,92	59,9		63,55	59,23	
			57,77	50,19	60,98	61,49		42,99	60,79	
			62,21	34,76	56,98	63,67		53,16	61,43	
			59,57	64,71	62,26	56,82		51,83	45,76	

			69,44<br>67,7<br>68,84<br>69,89<br>60,21<br>54,46<br>51,62<br>53,84<br>62,31<br>62,03<br>63,37<br>59,25<br>73,56<br>69,82<br>64,35<br>61,68<br>60,89<br>68,04<br>64,52<br>64,27<br>67,26<br>63,44<br>68,31<br>68,17<br>64,87<br>61,23	60,41<br>59,39<br>55,9<br>58,73<br>57,78<br>59,49<br>37<br>52,61<br>50,05<br>42,41<br>47,18<br>45,31<br>51,7<br>53,45	61,31<br>63,16<br>65,3<br>62,34<br>42,97<br>48,99<br>65,91<br>67,72<br>64,95<br>68,17<br>66,6<br>65,46<br>63,64<br>66,57<br>65,1<br>57,69<br>61,65<br>60,28<br>45,47<br>48,99<br>49,66<br>57,8<br>57,94<br>56,24<br>57,32<br>51,82<br>51,18<br>42,32	63,05<br>64,72<br>67,64<br>63,91<br>66,79<br>66,14<br>66,25<br>64,18<br>61,48<br>58,56<br>63,01<br>58,92<br>57,31<br>68,64<br>70,32<br>62,59<br>68,01<br>72,22<br>61,61<br>64,12<br>61,53<br>71,68<br>66,62<br>55,48<br>62,44<br>71,25<br>63,73<br>61,72<br>57,52<br>58,67<br>55,85<br>52,85<br>46,6<br>58,28<br>57,42<br>53,13<br>61,82<br>63,37<br>63,55<br>60,59<br>59,83<br>59,98<br>62,81<br>64,03<br>62,4<br>60,76<br>63,14<br>67,21<br>62,92<br>63,52<br>61,73<br>58,7<br>68,5<br>62,31		44,08<br>37,58<br>50,07<br>52,25<br>41,42<br>51,11<br>44,18<br>54,59<br>42,96<br>52,82<br>45,07	53,35<br>61,36<br>59,38<br>64,05<br>61,1<br>64,91<br>58,02<br>64,3<br>61,21<br>60,7<br>62,7<br>59,59<br>52,85<br>57,3<br>59,16<br>59,69<br>59,53<br>59,79<br>53,92<br>59,84<br>59,76<br>57,83<br>54,97<br>58,58<br>63,1<br>62,76<br>59,13<br>54,79<br>55,79<br>58,51<br>58,74<br>59,64<br>60,8<br>60,58<br>60,48<br>59,56<br>59,25<br>59,14<br>66,32<br>65,42<br>61,33<br>63,64<br>59,34<br>60,8<br>65,17<br>49,53<br>57,03<br>52,81<br>49,29<br>60,44<br>63,06<br>63,36<br>59,89<br>62,42

									64,56<br>60,23<br>60,86<br>60,99<br>66,02<br>63,93<br>63,7<br>63,74<br>64,97<br>63,21<br>67,66<br>66,42<br>67,01<br>65,18<br>58,04<br>63,62<br>58,88<br>59,37<br>61,98<br>61,34
**Mean α1**	**72,56±5, 35**	**50,2±7,4**	**65,118±4,89**	**56,41±8,22**	**57,085±7,1**	**63,43±4,70**	**58,0±10,8**	**59,5±7,5**	**60,7±3,8**

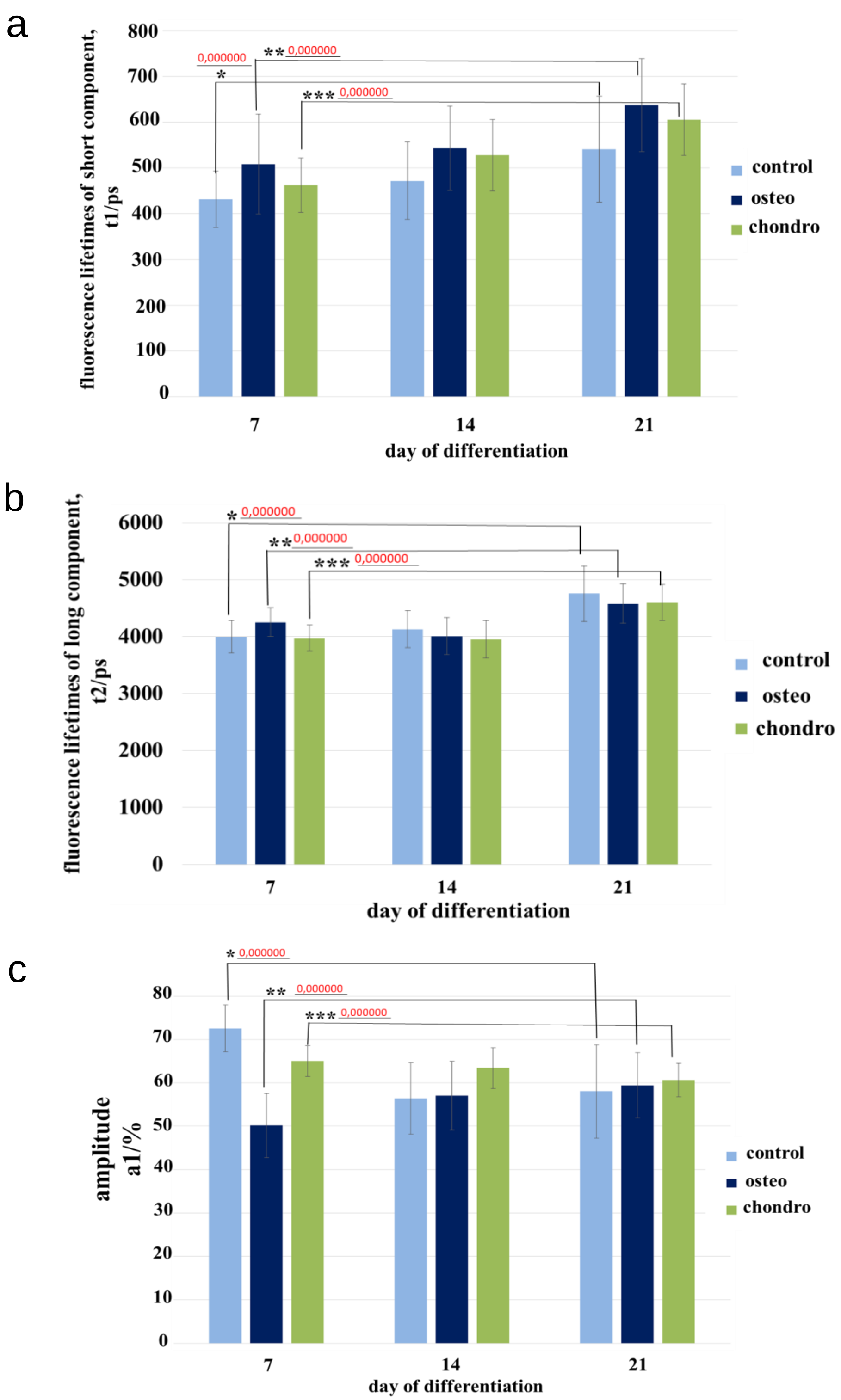

Figure S12. FLIM analysis of control MSCs, stained with BODIPY 1. Dynamics of the fluorescence lifetimes of the short, $\tau 1$, (a) and long, $\tau 2$, (b) components, and of the amplitude, $\alpha 1$ (c) in control and differentiated MSCs. Mean ± SD.

*Statistically significant difference with control MSCs on day 7

**Statistically significant difference with osteogenic differentiating MSCs on day 7

*** Statistically significant differences compared with chondrogenic differentiating MSCs on day 7.

In all examined locations at the plasma membrane BODIPY 1 had a biexponential decay with a short component ($\tau 1$) in the region of 500 ps (corresponding to ca 20-30 cP) and a long component ($\tau 2$) in the region of 4-5 ns (corresponding to several hundred cP up to 1500 cP).

The short component of these decays relates to a BODIPY orientation close to the head region of the bilayer, while the long component corresponds to the viscosity in the inner, highly hydrophobic tail region of the bilayer. The longer component ($\tau 2$) was converted to viscosity, as previously discussed (5, 6). α 1 and α 2 parameters were used to assess the correctness of the obtained data on fluorescence lifetimes and characterized the contribution of short component ($\tau 1$) and long component ($\tau 2$), which may reflect the distribution of BODIPY between two locations of the membrane, as previously discussed (5, 6).

Therefore, it should be noted that $\alpha 1$ and $\alpha 2$ parameters do not relate to the viscosity of the lipid tail region of the membrane, as $\tau 1$ and τaveraged are never converted to viscosities.

Undifferentiated cells: FLIM and mass spectrometry studies

We first performed FLIM measurements with BODIPY1 to compare undifferentiated cells on days 0-21 and differentiated MSCs on days 7-21. We observed a marked viscosity increase in all cell types with cultivation time: 1) undifferentiated day 21 cells were significantly more viscous than undifferentiated day 7 and 14 cells, and these were more viscous than undifferentiated day 0 cells. 2) osteogenically and chondrogenically differentiated cells on day 21 were more viscous than respected day 7 and 14 cells, and all were more viscous than undifferentiated day 0 cells. The fact that undifferentiated MSCs showed a significant increase in membrane viscosity on day 21 may be related to a significantly denser layer of MSCs.

However, the membrane viscosity rise in osteogenically and chondrogenically differentiated cell when compared to the undifferentiated day 0 cells may be associated with both an increase in confluence and a change in the membranes composition.

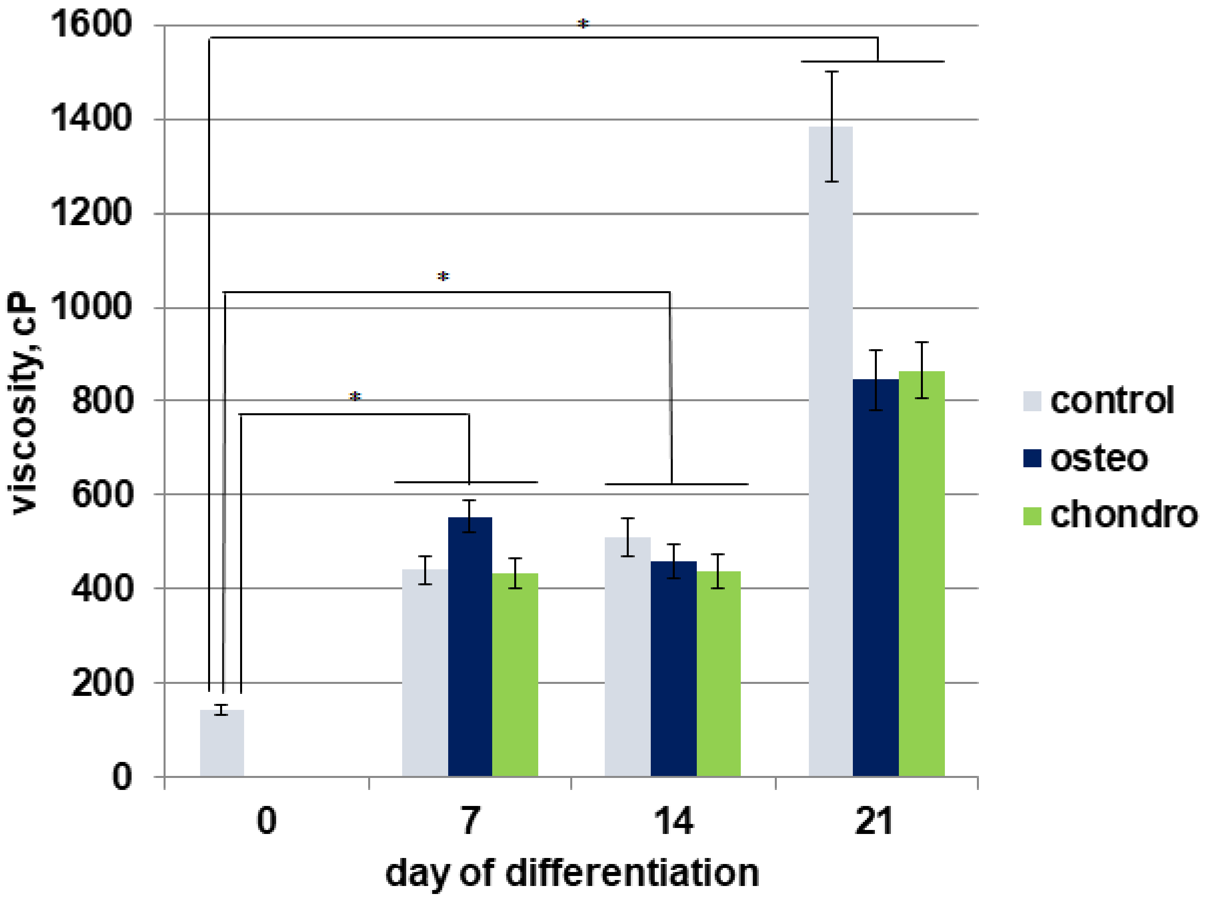

Figure S13. Viscosity analysis of MSCs lipid tail region via FLIM of molecular rotor BODIPY 1 during differentiation. Dynamics of the viscosity change during differentiation.* statistically significant differences compared with undifferentiated MSCs, p≤0, 05.

In order to gain insight on whether the lipid composition could cause the above viscsoity changes, we performed the time-of-flight secondary ion mass spectrometry on all cell samples. This is, however, a surface-sensitive technique, which requires dehydrated sample in vacuum conditions. It may be considered as a drawback in the context of our work since it is not possible to track changes in lipid composition over time for exactly the same cells. Nevertheless TOF-SIMS could provide valuable information comparing different cells or the same cell culture that is taken at different stages of the process (days 0, 7, 14, 21 in our case). Cell density on the substrate is the

main challenge in the analysis of this data, since lipid ion yield is proportional to the area occupied by cells.
ToF-SIMS analysis of cell culture required chemical fixation, rinsing and air drying as described in materials and methods section. The main aim of rinsing is removing of salts from the cell surfaces, since the excess of salts leads to organic ions signal suppression and dividing of a single species signal into multiple channels. However, we found that rinsing procedure, even when carefully controlled,   leads to loss of adhesion of some MSCs. The amount of cells removed from the substrate surface varies hence the protocol could not be considered as reproducible. The application of a layer of polylysine on the substrate solves this problem.
Polylysine layer does not affect data quantification for undifferentiated MSCs, osteogenic and hondrogenic differentiated MSCs on day 21 since the cells density on the substrates reaches its maximum (confluent cells). However, it severely complicates the analysis for growing undifferentiated MSCs with low density, due to the interference of lysine peaks from the substrate with peaks originating from cells. It is unclear what exact percentage of the examined area is occupied by cells and, hence, how to compare ion yields of differentiated MSCs at day 0 with others cells.
Nevetheless specific lipid ions could not originate from the substrate covered by polylysine. Hence, the ratio of these ions does not depend on cell density and can be compared. We used phosphatidylcholine peak (m/z 224) as a reference, which should be proportional to the surface cell coverage and is not affected by polylysine signal. Fig. S14 shows that SM/PC and cholesterol/PC ratios significantly differs for undifferential cells on day 0 and day 21. Interestingly, the ratios change in the opposite direction. While SM/PC ratio decreases for undifferentiated MSCs day 21 compare to day 0, cholesterol/PC ratio increases. A high level of sphingomyelin in undifferentiated MSCs at day 0 may be associated with their high metabolic activity. These cells actively proliferate, that is associated with the activation of signaling pathways. Sphingomyelin is known to play an important role in cell signaling (7).

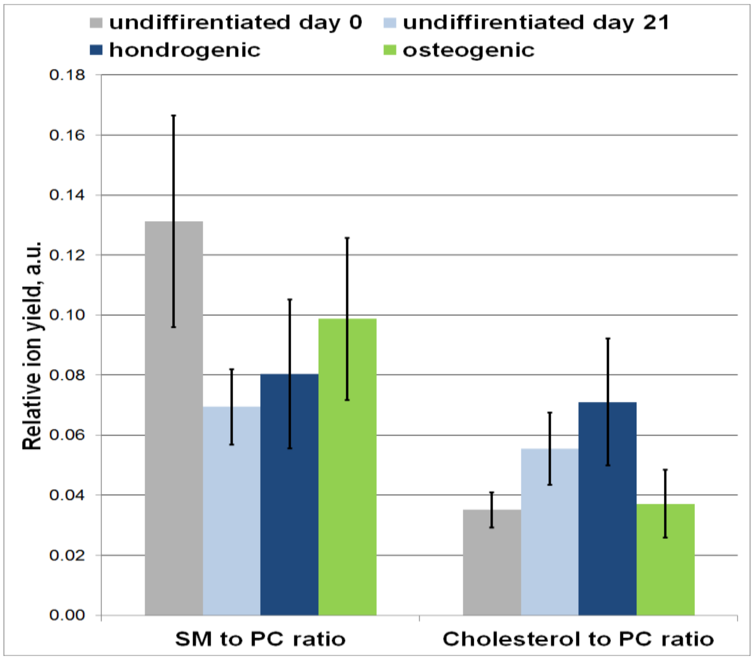

Figure S14. Lipid analysis by ToF-SIMS. Relative ion yield of sphingomyelin and cholesterol relative to phosphatidylcholine ion yield.

Fatty acids analysis (Fig. S15) does not reveal significant differences in saturated and overall unsaturated fatty acids yields. A significant decrease in amount of polyunsaturated fatty acids is

clearly observed for undifferentiated MSCs on day 21 compare to day 0. Moreover, chondrogenically and osteogenically differentiated cells also show lower relative PUFAs ion yield. The decrease in PUFAs relative ion yield between undifferentiated cells day 0 and day 21 is in agreement with viscosity measurements provided by FLIM.

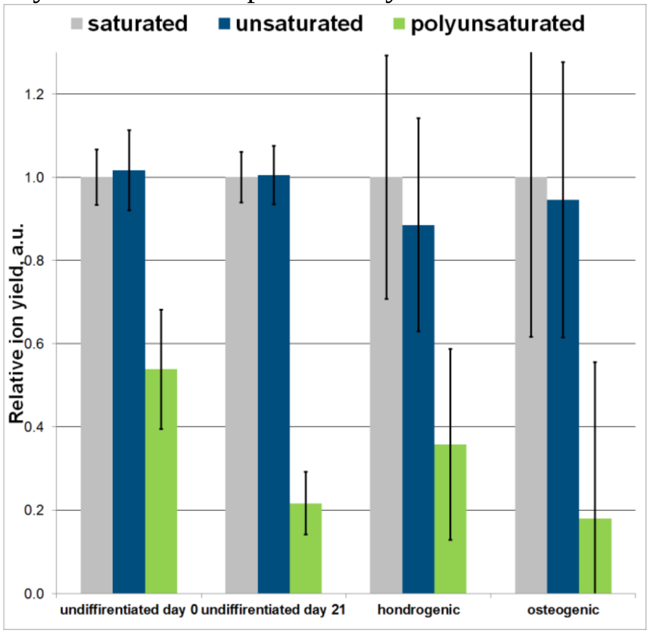

Figure S15. Lipid analysis by ToF-SIMS. Fatty acids analysis. Ion yields are normalized to ion yield of saturated fatty acids.

Thus, these data showed that changes in the viscosity of the membrane in cells on day 21 can be caused by changes in membrane lipids.

References

1. Ahmed, A. N., Gulera, E., Ucana, H. I, Boyleb, R. W. *Dyes Pigm*. **94**, 496 (2012).
2. Shirmanova, M. V., Shimolina, L. E., Lukina, M. M., Zagaynova, E. V., Kuimova, M. K. Live Cell Imaging of Viscosity in 3D Tumour Cell Models. In: Dmitriev R. (eds) Multi-Parametric Live Cell Microscopy of 3D Tissue Models. *Adv Exp Med Biol*. **1035**, 143-153 (2017).
3. Shimolina, L. E., Izquierdo, M. A., López-Duarte, I., Bull, J. A., Shirmanova, M. V., Klapshina, L. G., Zagaynova, E. V., Kuimova, M. K. Imaging tumor microscopic viscosity in vivo using molecular rotors. *Scientific Reports* **7**, (2017).
4. Wu, Y., Stefl, M., Olzyńska, A., Hof, M., Yahioglu, G., Yip, P., Casey, D. R., Ces, O., Humpolíčková, J., Kuimova, M. K. Molecular rheometry: direct determination of viscosity in Lo and Ld lipid phases via fluorescence lifetime imaging. *Phys. Chem. Chem. Phys* **15**, 14986–93 (2013).
5. Dent, M. R., López-Duarte, I., Dickson, C. J., Geoghegan, N. D., Cooper, J. M., Gould, I. R., Krams, R., Bull, J. A., Brooks, N. J., Kuimova, M. K., Imaging phase separation in model lipid membranes through the use of BODIPY based molecular rotors. *Phys. Chem. Chem. Phys.* **17**, 18393–18402 (2015).
6. Sherin, P. S., López-Duarte, I., Dent, M. R., Kubánková, M., Vyšniauskas, A., Bull, J. A., Reshetnikova, E. S., Klymchenko, A. S., Tsentalovich, Y. P., Kuimova, M. K. Visualising the membrane viscosity of porcine eye lens cells using molecular rotors. *Chem Sci* **8**, 3523-3528 (2017).

7. Kolesnick, R. Signal transduction through the sphingomyelin pathway. *Molecular and chemical neuropathology* **21**, 287–297 (1994).
